# mRNA Display for Identifying Peptide Substrates of Enzymes

**DOI:** 10.1021/acs.biochem.6c00101

**Published:** 2026-06-08

**Authors:** Georgi M. Trifonov, Ella C. Sames, Tom E. McAllister

**Affiliations:** Chemistry, School of Natural and Environmental Sciences, 5994 Newcastle University, Newcastle Upon Tyne, Tyne and Wear NE1 7RU, U.K.

**Keywords:** substrate identification, mRNA-display, cDNA-display, proteases, post-translational modifications, RiPPs, in vitro translation

## Abstract

Among display technologies, mRNA display has emerged as a powerful approach to identify *de novo* ligands for proteins of interest. Applying the same methods to studying the substrates of protein/peptide-modifying enzymes has received much less attention, but progress in this area has accelerated rapidly over the last 5 years. In this article, we review the published literature to date (up to December 2025) of dedicated efforts to identify and understand enzyme substrate preferences using mRNA- or cDNA-display. We include observations of trends from these reports over time and reflections on where the area may go in the future. We hope this will serve as a useful primer for researchers in this and related areas.

## Graphical abstract

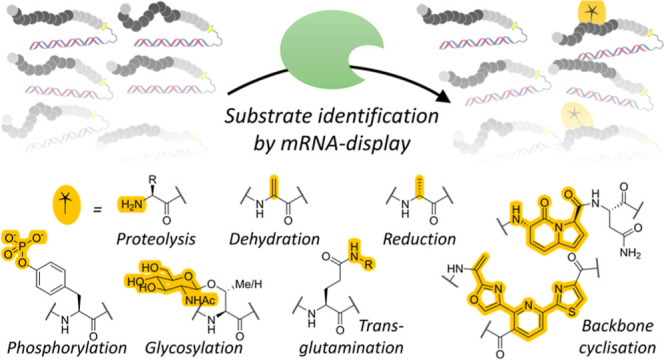

## Introduction

Display technologies have emerged as powerful methods for screening large numbers of peptides for desirable characteristics, typically binding to a protein target. Phage display is the most well-established, with the first report of antigen display on phage particles published in 1985,^1^ and half of the 2018 Nobel Prize in Chemistry awarded to George P. Smith and Sir Gregory P. Winter “for the phage display of peptides and antibodies”.^2^ Many alternative display technologies^3^ have since emerged, including bacteria^4,5^ and yeast cell–surface display,^6,7^ and cell-free approaches, such as ribosome display^8,9^ or mRNA-display.^10^

Screening campaigns ordinarily consist of iterative rounds of affinity enrichment through binding to an immobilized protein target. The largest application of these display technologies is for identifying binding ligands, or more specifically inhibitors for therapeutically relevant targets (though there is no guarantee that a ligand will be inhibitory), and these can be based on existing scaffolds (e.g., antibodies or parts thereof) or novel modalities such as cyclic peptides. The peptidic nature of the ligand could perhaps be viewed as a technical requirement rather than a desirable characteristic for inhibitor discovery; an inconvenient necessity for exploiting the power of templated ribosomal translation to screen and subsequently deconvolute vast libraries, in the order of trillions of distinct molecules. Simple peptides are not promising clinical candidates, usually with short plasma half-life and poor passive membrane permeability^11^ though there are examples where these hurdles have been overcome.^12^ The invention of DNA-encoded libraries in the early 2000s addresses exactly this point; retaining the power to screen large libraries and deconvolute by DNA sequencing without the requirement for ribosomal synthesis, but comes with its own challenges.^13^ Innovative approaches have been adopted to adapt display techniques to improve the prospects of peptides as drugs, most notably codon expansion/reprogramming. This is either achieved by replacement of “natural” (i.e., proteinogenic) amino acids (nAAs) with analogues that can be used by aminoacyl-tRNA synthetases^14^ to transfer the analogue to a corresponding tRNA or prior acylation of tRNAs by flexizymes. Flexizymes enable acylation with potentially any carboxylic acid, unconfined by similarity requirements to nAAs (though it must be noted that, even once charged, amino acids may prove incompatible with the subsequent steps of ribosomal translation). A full discussion of this area is beyond the scope of this review, and we refer the reader to other texts for more information.^15–17^

In this article, we will concentrate on concerted efforts to identify enzyme substrates using in vitro display technologies in which enzymatic modification of the peptide is an essential part of the enrichment process.^18^ This excludes the identification of ligands that only bind and are not enzymatically modified by the enzyme studied. The most straightforward application for substrate identification is if an enzyme acts upon an unmodified, native peptide. Native peptide display can be used to gain information on the addition of post-translational modifications (PTMs), such as glycosylation, phosphorylation, acetylation, or methylation; peptide cleavage (e.g., proteases); or ligation with another peptide substrate. A key advantage of mRNA-display (and other in vitro display methods) is the capability to go beyond nAAs and incorporate unnatural amino acids (UAAs–specifically nonproteinogenic AAs), e.g., post-translationally modified amino acids (PTMs), in a templated manner, thus enabling dissection of binding interactions requiring combinations of modifications or when the addition of one modification is dependent upon the presence of another, such as in histone tails,^19^ ubiquitin,^20^ or *O*-glycoproteins.^21^

There are numerous similarities but some key differences in the approach taken to substrate identification rather than ligand discovery. The initial steps are analogous: a DNA library is prepared, transcribed into RNA and ligated to an appropriate puromycin-containing oligonucleotide before translation (optionally including UAAs) to generate peptides covalently linked to their encoding mRNA strand, and reverse transcription is conducted to generate cDNA. In mRNA display, peptides are linked to cDNA via complex formation with mRNA and screened as peptide-mRNA/cDNA complexes (Figure 1). In cDNA display, the cDNA strand is primed from the puromycin oligonucleotide, so it is directly covalently linked to the peptide; this may then be screened as a peptide-cDNA/mRNA complex or the mRNA removed and peptides screened as peptide-cDNA complexes. We do not draw a strong distinction between these approaches for the purposes of this review. Unlike ligand discovery efforts, where enrichment of “hit” sequences occurs through binding to the target, modified peptides will not remain bound to the enzyme, so a further capture step is required, which is bespoke to each enzyme investigated. Following capture to separate modified and unmodified sequences, the cDNA is recovered and then amplified by PCR to create the input library for the subsequent round and/or analyzed by next-generation sequencing (NGS). Pre- and/or post-enzymatic modification steps may also be used, typically to facilitate differentiation of substrates and non-substrates for the capture step. These may be chemical and/or enzymatic and form a crucial part of the substrate identification workflow.

**Figure 1:**
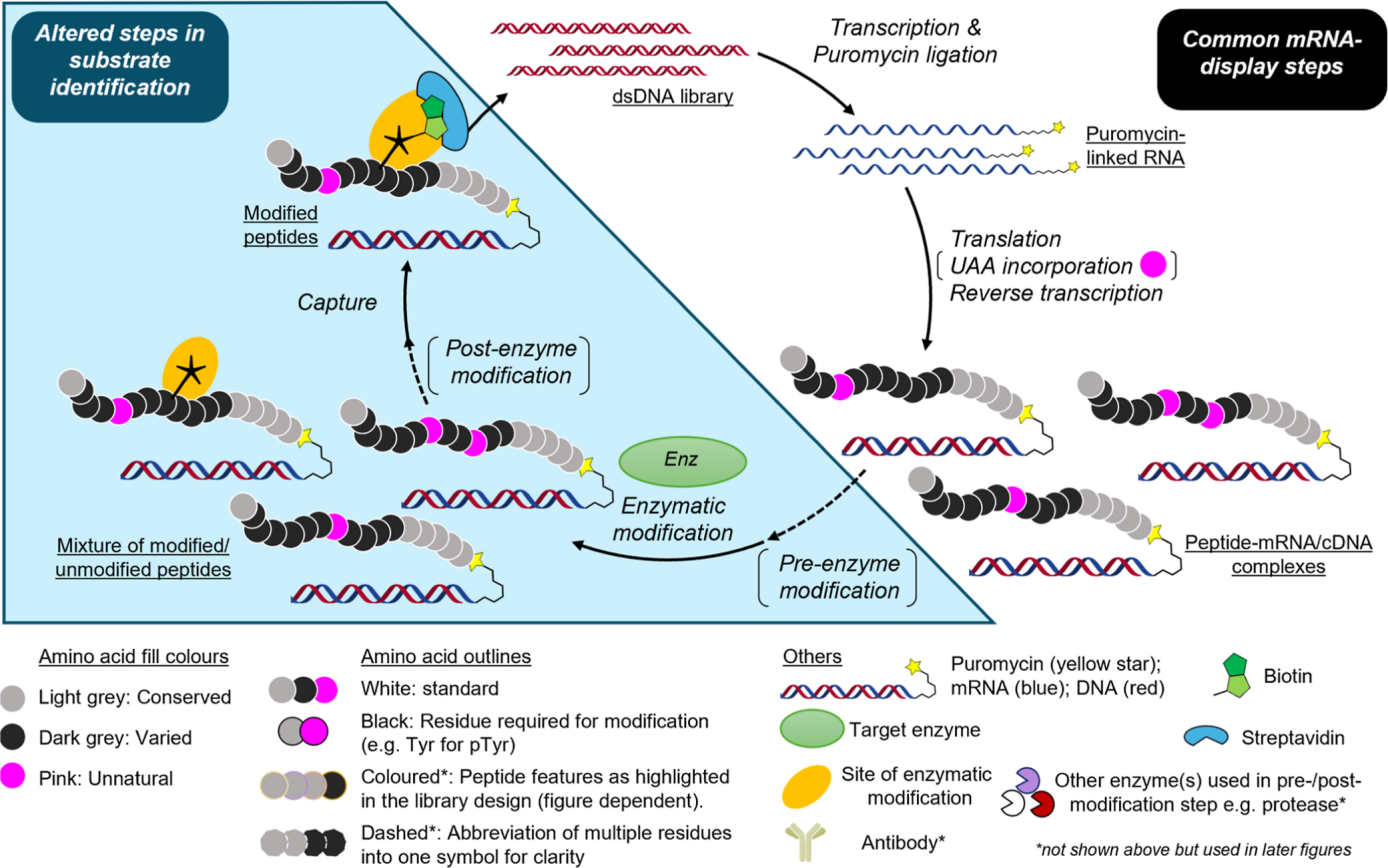
mRNA-display selection cycle with specific steps for substrate identification highlighted in the blue region and optional steps shown in parentheses and with dashed arrows. Variable amino acids are shown as black circles, UAAs as pink circles, conserved regions (e.g., for purification tags, etc.) as gray circles, and puromycin as a yellow star. The orange oval represents the enzymatic modification of interest, here showing addition of a group, and the green pentagons a purification handle (e.g., biotin). A key to symbols used throughout the figures in this review is included.

## Examples from Literature

We identified 24 reports in the literature meeting our criteria of enzymatic modification of mRNA/cDNA-display peptides to investigate substrate specificity, making such reports much less frequent than reports of de novo cyclic peptide ligands, for example (where ∼1 paper has been published every 1.5 months since 2015),^22,23^ and even serendipitous discovery during ligand identification seems very uncommon. We are aware of one report of inadvertent substrate discovery, by Kawamura et al.,^24^ when an inhibitory cyclic peptide could be converted into a substrate by rational modification. The lack of similar reports is perhaps not surprising; inhibitor discovery is the aim of most campaigns, and thus exploration of hits as substrates is likely a secondary concern at best. The presence of modifiable residues within de novo ligands is no guarantee of substrate activity, as was the case for proline-containing peptide ligands identified for prolyl hydroxylase 2.^25^ Unless actively seeking out modified peptides, these could easily be removed during the selection process or, even if enriched, not be subsequently prioritized for further characterization in favor of other, more potent ligands.

Each identified report (Table 1) is covered in greater detail in the section below. We include details of the enzyme(s) investigated, library designs, and sequence diversity, as well as the experimental steps specific to substrate identification (blue area of Figure 1), i.e., enzymatic modification, pre- and/or post-enzymatic modification(s), and capture, as well as a brief summary of the findings. We have grouped these according to the type of enzyme investigated: PTMs, 4 articles; proteases, 10; transglutaminases, 4; and ribosomally synthesized, post-translationally modified peptide (RiPP) synthetases, 6 (N.B. one article reports multiple classes of enzyme and is covered in both the PTMs and proteases sections).

**Table 1:** Previous Literature Reports of Enzyme Substrate Identification by mRNA-Display^a^

Enzyme type	Enzyme studied and reference	Pre-enzyme modification(s)	Post-enzyme modification(s)	Capture
PTM	Abelson murine leukemia virus tyrosine kinase (v-Abl kinase)^26^	N	N	Ab
	*Homo sapiens* tyrosine kinase ABL1 (Abl kinase)^27^	Y	N	Ab
	*Homo sapiens* *O*-GlcNAc (*N*-acetylglucosamine) transferase (OGT)^28^	N	N	Ab
	*Bacillus megaterium* tyrosinase (megTYR)^29^	N	Y	Bt
Protease	*Homo sapiens* caspase-3, caspase-8, and granzyme B^30^	Y	N	Cov^i^
	Hepatitis C virus NS3/4A protease (HCV NS3/4A)^27,31^	Y	N	Bt
	*Homo sapiens* furin^32^	Y	N	Bt
	*Bacteroides fragilis* metalloprotease II (MPII) and fragilysin 3 (FRA3)^33,34^	Y	N	Bt
	*Homo sapiens* matrix metalloproteases (MMPs)^35^	Y	N	Bt
	*Homo sapiens* Chymotrypsin^36^	N	N	Bt
	*Homo sapiens* factor Xa (factor Xa),*Homo sapiens* disintegrin and metalloprotease 17 (ADAM17),*Streptomyces pyogenes*cysteine protease/SpeB (streptopain)^37^	N	N	Cov^i^
	Methionine amino peptidase (MAP)^38^	Y	Y	Bt
Transglutaminase	*Streptomyces mobaraensis* transglutaminase (STG*)^39^	N	N	Cov^ii^
	*Homo sapiens* transglutaminase 2 (TG2)^40^	N	N	Bt
	Microbial transglutaminase from*Streptomyces mobaraensis* (mTG*)^41^	N	N	Bt
	*Homo sapiens* transglutaminase 1 (TG1)^42^	N	N	Bt
RiPP	Pantocin A synthase (PaaA) from*Pantoea agglomerans* ^43^	N	Y	Bt
	Lactazole biosynthetic enzymes (LazBCDEF) from *Streptomyces lactacystinaeus* ^44^	N	N	Bt & HA
	Lactazole biosynthetic enzymes (LazBF and LazDEF) from *Streptomyces lactacystinaeus* ^45^	N	Y	Bt & HA
	Cyclodehydratase (LynD) from *Lyngbya aestuarii* strain PCC 8106 ^46^	N	Y	Bt
	LanJ_C_ dehydroamino acid reductases from multiple organisms^47^	Y	Y	Bt & HA
	*Flavobacterium terrae* dehydroamino acid reductase (FltJ_A_)^48^	Y	Y	Bt & HA

a Ab–antibody to modification, Bt–biotin, Cov–covalent attachment (i: library immobilized prior to enzyme treatment; ii: action of enzyme forms covalent linkage to immobilized substrate), and HA–Hemagglutinin tag (YPYDVPDYA). *STG and mTG appear to be the same protein given different names in different publications.

### Post-Translational Modifications

Of the four papers in this section, three report PTMs where additional groups are introduced (phosphorylation or glycosylation), and capture is achieved by antibodies specific for the modification. This use of antibodies appears to be restricted to studying PTMs and requires consideration of any underlying epitope preferences of the antibody itself, which could influence the substrates identified. The fourth article within this section concerns tyrosinase, which converts the phenol group of tyrosine into an ortho-quinone. While not an endogenous PTM to our knowledge, we include this here as the activity is not obviously part of the other groups and involves the modification of an amino acid within a peptide.

In 2002, the first report of substrate identification by mRNA-display that we are aware of, Cujec et al. determined the identity of the peptide substrates of the Abelson murine leukemia virus tyrosine kinase (v-Abl kinase), a protein highly homologous to the human C-Abl kinase, both known to be oncogenes in their respective organisms.^26^ An initial screen used a randomized peptide library containing a conserved central Tyr residue flanked on both sides by five randomized amino acids encoded by NNS codons (theoretical diversity 1 × 10^13^) and constant N- and C-terminal sequences each containing a Cys residue (Figure 2). The authors note that these cysteines could be used to constrain the random region as part of a loop, but this was not done in this work.

**Figure 2:**
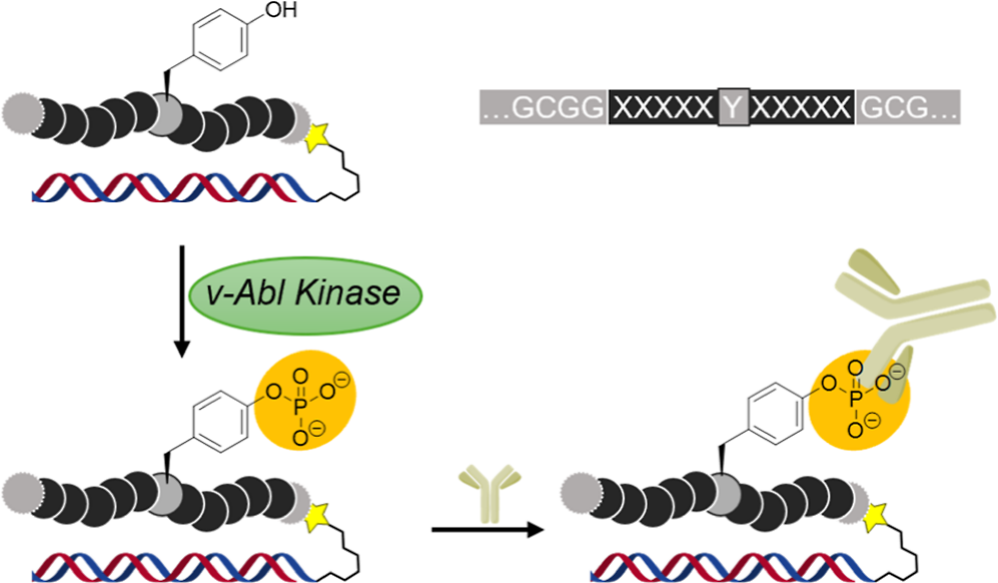
A library comprising 5 randomized amino acids flanking an invariant tyrosine residue, X_5_-Y-X_5_, was incubated with v-Abl kinase and phosphorylated peptides captured were by anti-phosphotyrosine antibody.

The library was incubated with v-Abl kinase, and phosphorylated substrates were captured with a phosphotyrosine-specific antibody (α4G10). After six rounds of selection, the enriched peptides were sequenced by transformation into *Escherichia coli* cells, and 69 individual colonies were sequenced by chain-termination sequencing. We note that at the time of this work, massively parallel sequencing approaches were not well established, and as a result, the level of detail attainable from this level of sequencing is significantly reduced. Most (46/69) matched the established substrate sequence motif of I/L/V–Y-X_1–5_-P/F and almost all (66/69) contained an additional Tyr residue within the randomized region. Two clones no longer contained the conserved central Tyr, one of which did instead contain a Tyr within the variable region. Overall, there was no clear consensus sequence from this randomized peptide library, so to gain insight into cellular targets of v-Abl kinase, a second selection procedure was undertaken using a library of mRNA templates derived from human bone marrow cells. Results from this cellular selection also identified several substrates of v-Abl previously identified in vivo, but also novel targets such as the SH2 domain-containing Shg protein. In addition, substrates from the cellular mRNA-protein libraries had sequences closely matching those identified in the randomized peptide library screen.

Subsequently, Kozlov et al. also performed a selection using a tyrosine kinase, the *Homo sapiens* Abl kinase (also known as C-Abl).^27^ This used a designed 3243-member library of 10-mer peptides containing known substrate sequences of various tyrosine kinases, derivatives thereof, and negative controls without Tyr residues. All peptides had an N-terminal TEV protease site (GENLYFQ↓CA) used in a pre-enzyme modification step to generate an N-terminal Cys that was subsequently biotinylated using a biotin-PEG thioester (Figure 3). The eluted cDNA-peptide conjugates were incubated with Abl kinase before purification of the library via streptavidin immobilization and release, and then substrates were captured in a step with anti-phosphotyrosine antibody.

**Figure 3:**
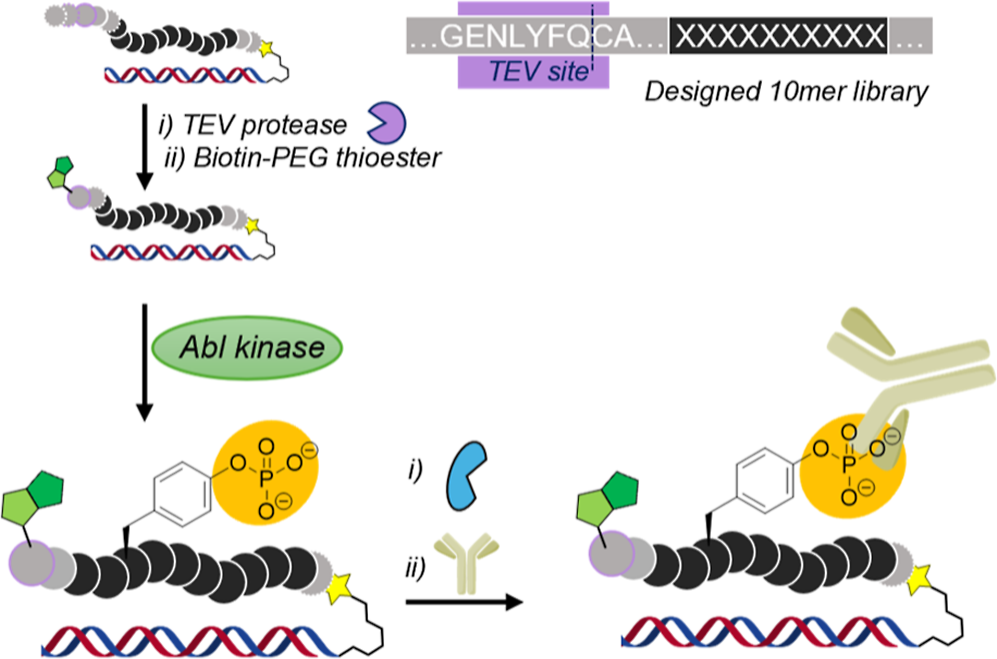
Identification of peptide substrates of Abl kinase using a curated 3243-member 10-mer starting library. Pre-enzymatic modification steps used TEV protease to generate an *N*-terminal Cys that was reacted with biotin thioester. Following incubation with Abl kinase, phosphorylated peptides were captured using an anti-phosphotyrosine antibody. From the information available, we were unable to determine the specific chemical structure of the biotin thioester used; the green pentagons are used to represent the biotin moiety (as in other figures) with an unknown linkage to the peptides.

After a single round of selection, over 90% of the known substrate group (GEAIYAAPFA and every individual single point mutant except Tyr5, 198 peptides) within the pool were identified, as well as 4% of the 1355 sequences with at least 1 tyrosine. The sequence preferences inferred from the GEAIYAAPFA set were in line with those previously established.

Beyond kinases, Shi et al. used mRNA-display to study protein *O*-GlcNAc, a modification of Ser/Thr/Tyr residues, again using an antibody against the installed modification to capture modified peptides.^28^ The library comprised an initiating formyl Met, nine variable residues encoded by NNK codons and a (Gly–Ala)_3_ spacer. The theoretical diversity of this library is 5.12 × 10^11^, but the stated achieved diversity was 5.12 × 10^9^, noting that at these large library sizes, often the reaction scale becomes a limiting factor, i.e., there is a single molecule corresponding to a given sequence at the outset. Four rounds of modification with human *O*-GlcNAc transferase (OGT) were performed, including preclear steps each time (incubation of unmodified library with the immobilized antibody to remove peptides with inherent antibody binding capability) before capturing modified peptides using the RL2 anti-*O*-GlcNAc antibody (Figure 4). Enriched sequences were ligated and transformed into *E. coli* before individual colonies were sequenced; 14 of 16 unique peptides contained Ser/Thr residues (thought to be the main acceptor sites for O-GlcNAcylation by OGT), but upon resynthesis and follow-up assays, none proved to be substrates.

**Figure 4:**
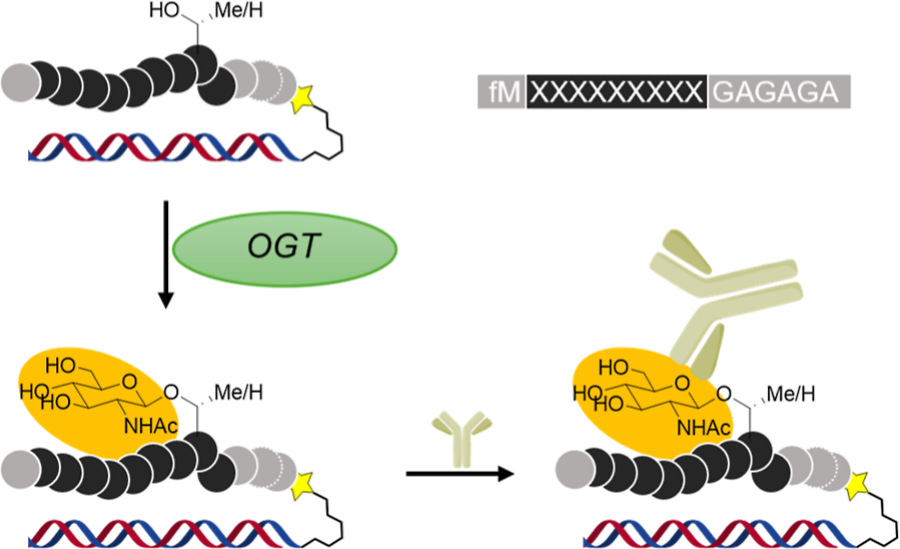
A random X_9_ library was prepared to identify substrates of OGT with modified (GlcNAc-ylated) peptides captured by RL2 anti-*O*-GlcNAc antibody.

Subsequent deep sequencing of earlier rounds showed the libraries remained highly diverse and provided little further insight except for the occurrence of an unexpected peptide, RESSYDIYRVPSSQS (termed ZO-3), too long to originate from the X_9_ library and previously used as a positive control for optimization of the approach. The authors suggest this entered the selection as a contaminant, even though precautions against this were taken. In their earlier experiments, modest enrichment (∼4-fold) of ZO-3 was observed after OGT treatment compared to no treatment, which was attributed to the acceptor site’s position near the C-terminus (underlined above) in proximity to the puromycin linker that could lead to inhibition. To test this hypothesis, a variant of ZO-3 with N-terminal linkage to a dsDNA oligonucleotide was prepared. This had a much shorter linker between peptide and oligonucleotide: ∼12 atoms in the “N-terminal display” variant compared with ∼100 atoms with the puromycin linker oligo used in the mRNA-display, so it is not a direct comparison. Nevertheless, the N-terminal ZO-3 showed far higher recovery with the RL2 antibody after incubation with OGT than without (∼2400-fold). The authors conclude that the C-terminal position of the mRNA attachment in the mRNA-display library was preventing substrate modification, an important finding and an inherent limitation of the mRNA-display approach. Another highlighted shortcoming is the potential for bias arising from the antibody’s epitope preferences, and several alternative approaches to overcome this are suggested, including the use of azido-modified UDP-GlcNAc substrates to directly install an azide moiety by the action of OGT or subsequent modification of the installed GlcNAc to add an alternative purification handle; the latter approach is seen in many other reports (vide infra).

For the discovery of de novo peptide ligands with high-affinity binding conformations, mRNA display is commonly combined with a variety of peptide cyclization strategies. However, using enzymes to affect cyclization carries a risk of introducing bias to any subsequent selection. To investigate the potential to use a tyrosinase to effect peptide cyclization, Fleming et al. used mRNA display to characterize substrate preferences of *Bacillus megaterium* tyrosinase (megTYR).^29^ To this end, a library composed of a formyl Met initiator, invariant tyrosine and 4-mer NNK encoded randomized amino acid region (total diversity 16,000), was exposed to megTYR, converting the phenol group to an ortho-quinone. In this case, an in situ modification step was performed by the inclusion of biotin-cysteamine during the megTYR reaction; the thiol of biotin-cysteamine selectively reacts at C5 (and, to a lesser extent, C2) of the ortho-quinone generated. Modified peptides could then be captured with streptavidin beads (Figure 5).

**Figure 5:**
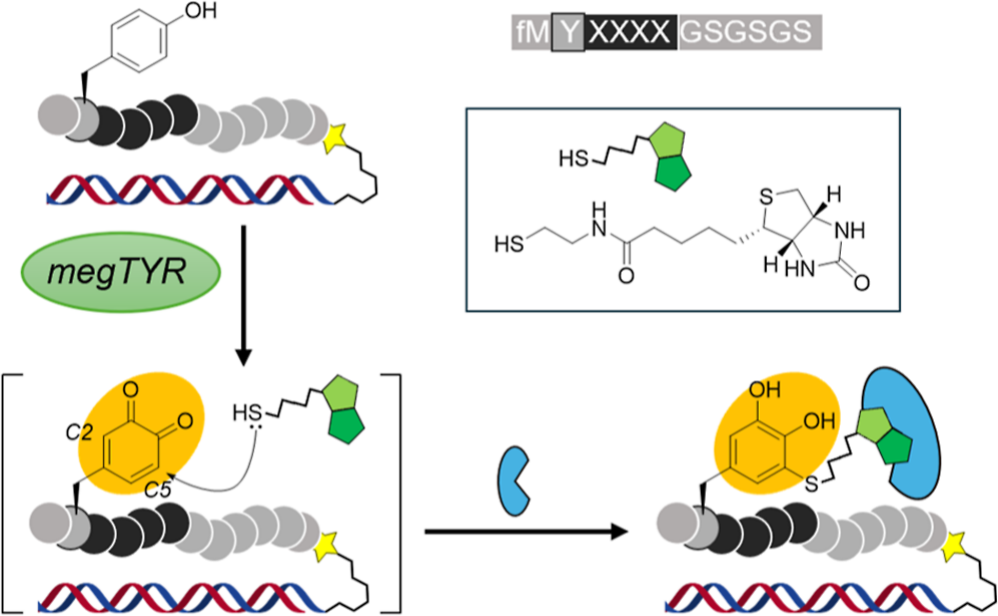
Tyrosinase activity was screened against substrates with 4 residues C-terminal to Tyr randomized. Modified substrates were captured by streptavidin, following in situ modification of product peptides with biotin-cysteamine.

A single round of selection was conducted in triplicate for each of 6 enzyme concentrations (2 μM–0.125 μM, 2-fold dilutions), showing that near quantitative recovery was achieved for megTYR concentrations of 1 μM or above in a 1 h reaction time. Reasoning that lower recovery would be more informative for substrate preferences, the 0.125 μM selection was sequenced (∼50% total recovery). Analysis showed little position-wise effects, indicative of broad specificity, useful in this application for generating cyclic peptides. Pro-containing peptides were poor substrates, with the effect strongest directly next to the invariant Tyr and decreasing further away. Asn, Gln, Glu, and particularly Asp were enriched throughout, but startlingly, Cys-containing substrates were highly depleted. This was attributed to preferential intramolecular thiol attack from the Cys side chain outcompeting the intermolecular reaction from biotin-cysteamine leading to inefficient capture, which is promising for generating unbiased libraries of cyclic peptides. This was demonstrated later in the article with the identification of a potent tyrosinase-cyclized peptide inhibitor of melanoma-associated antigen A4 (MAGE-A4).^29^

Overall, these 4 papers demonstrate the advances made in display technologies and the variety of approaches that can be taken to library generation and reactivity screening. Some of the pitfalls of the approach are also highlighted; use of antibodies as capture agents may introduce their own epitope bias to the results, and it is important to remember that the appended encoding oligonucleotides may not be benign and can influence the selection. It is likely that this is a much more common occurrence than is suggested by reports in the literature, as “failed selections” are not easily publishable.

### Proteases

Reports of understanding protease specificity are the most abundant, perhaps as display approaches can be readily applied to such enzymes; immobilization at the N-terminus gives an easy read-out for peptide backbone cleavage and facile separation.

The first report investigating proteases was by Ju et al. in 2007.^30^ Caspases are a family of proteolytic enzymes that are crucial in mediating the pathway of apoptosis; however, the specific mechanisms that caspase activation facilitates that lead to cell death are unknown. mRNA-display was used to understand the natural substrates of the Caspases by employing a library representative of the human proteome. Briefly, a mixture of Poly­(A)^+^ mRNAs from human brain, heart, spleen, thymus, and muscle, cDNAs were generated and amplified by PCR with primers to introduce the features required for mRNA-display; upstream T7 promoter and a downstream puromycin ligation site. This also introduced an N-terminal AviTag and a C-terminal FLAG or His_6_ tag to the coding region (Figure 6a), and the PCR product was fractionated to achieve peptide sequences of 80–200 amino acids in length, which the authors note may be sufficiently long to adopt natural confirmations or even tertiary structures. In this case, the diversity of the library is unknown but has an upper bound of 9 × 10^11^ (1.5 pmol of mRNA used in each round).

**Figure 6:**
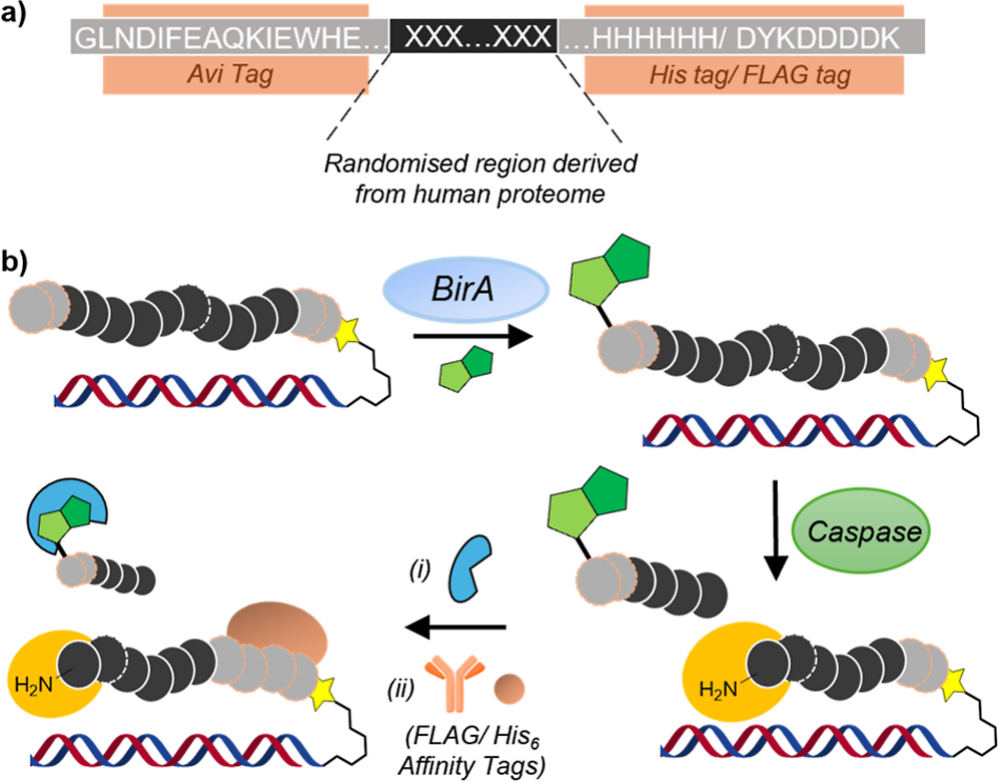
mRNA-display workflow for caspase substrate identification; (a) the library design comprised mRNA from various tissues, so it is thus expected to be representative of the human proteome, flanked with an N-terminal AviTag sequence, and C-terminal His-tag or FLAG tag; (b) the selection process utilized BirA in a pre-modification step to load biotin onto Lys within the AviTag. Incubation with a caspase cleaves peptide substrates. The C-terminal residues are isolated in step (i) by pull-down with streptavidin-coated magnetic beads (blue truncated disk), and the N-terminal residues are separated in step (ii) with FLAG/His_6_ affinity tags (orange antibody/brown sphere) and the fused mRNA is sequenced.

In a pre-enzymatic modification step, the AviTag was biotinylated using BirA, allowing peptides to be immobilized onto streptavidin-agarose beads. Incubation with the protease then cleaves substrates, releasing the *C*-terminal fragment into solution attached to the encoding mRNA. This fragment is captured by either FLAG or His_6_ affinity tags, and the attached mRNA is amplified by PCR and used for subsequent rounds (Figure 6b).

For caspase-3, three rounds of selection were performed, and sequences were identified by ligation into a vector and transformation into *E. coli*. To enable identification of less enriched substrates, the 12 most abundant sequences were removed by hybridization to complementary oligonucleotides, and sequencing was repeated. In total, this identified 207 unique proteins from 580 clones, and 115 were confirmed as caspase-3 substrates, 89 of which were novel and contained the established DXXD motif. A similar process was used for caspase-8, and follow-up work unraveled the proportion of substrates that were cleaved by only caspase-3, only caspase-8, or both. In a further experiment, granzyme B (a protease from outside the caspase family) was investigated. To identify non-caspase substrates for granzyme B, the library was first incubated with a mixture of seven caspases (2, 3, and 6–10), washed to remove cleaved sequences, and then incubated with granzyme B, identifying >60 substrates.

Reported in the same manuscript as the Abl kinase discussed previously, Kozlov et al. investigated the potential cleavage sites of the hepatitus C virus (HCV) NS3/4A protease using a library of 3001 peptides comprising 10-mer overlapping sequences covering the entire translated genome of the virus (Figure 7a,b).^27^ As previously described in the PTM’s section, all peptides had an N-terminal TEV protease site GENLYFQ↓CA used in a pre-enzyme modification step to generate an N-terminal Cys that was subsequently biotinylated using a biotin-PEG thioester, and the library was immobilized on streptavidin-coated beads (Figure 7a). Bead-bound peptides were incubated with HCV NS3/4A, with cleavage releasing the C-terminal peptide/cDNA conjugate from the bead and uncleaved sequences remaining bound. The screen identified three previously known cleavage sites (^1708^MEEC↓SQHL^1715^, ^1969^TTPC↓SGSW^1976^, and ^2417^VVCC↓SMSY^2424^) and two previously unknown cleavage sites (^2168^VAVLT↓SMLTD^2177^ and ^672^QVLPC↓SFTTL^681^). However, the authors note that these novel sites are most likely not biologically relevant as they correspond to internal positions and would be inaccessible to the protease in the folded state, highlighting a potential disadvantage of the peptide display approach, where sequences typically lack secondary structures.

**Figure 7:**
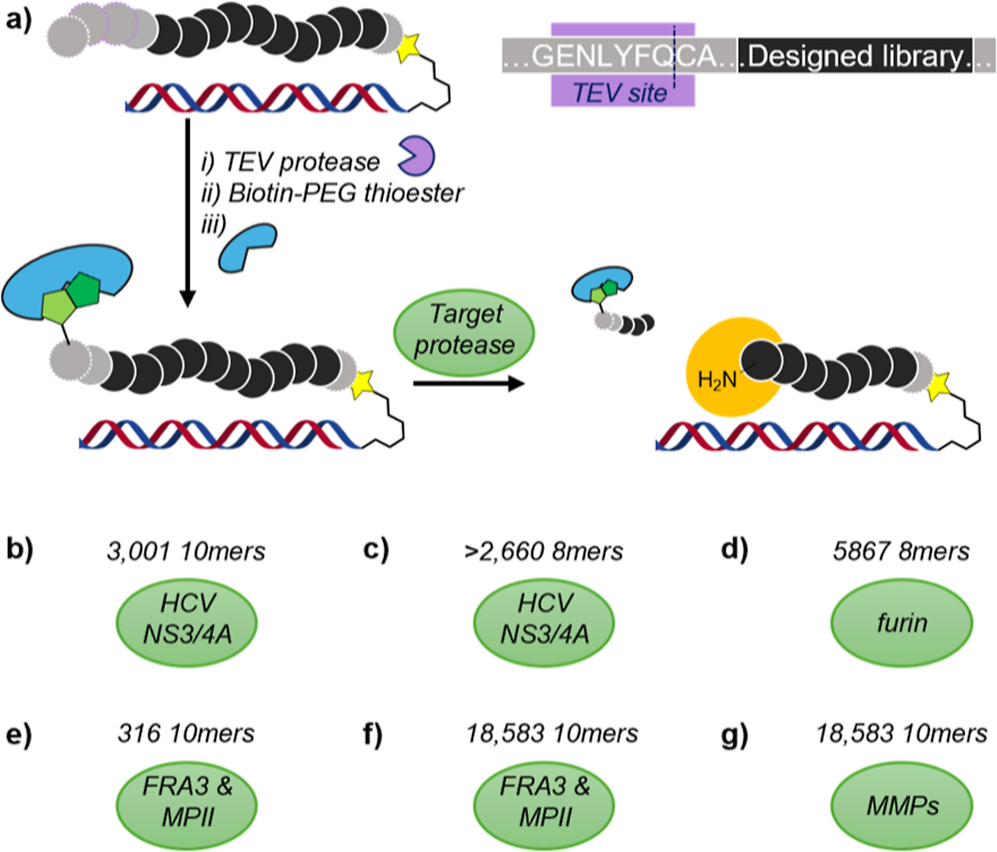
(a) Library design and selection overview of the reports from the group at Prognosys Bioscience. From the information available, we were unable to determine the specific chemical structure of the biotin thioester used; the green pentagons are used to represent the biotin moiety (as in other figures) with unknown linkage to the peptides. Proteases targets, library lengths, and diversity are noted in (b–g); (b) HCV NS3/4A;^27^ (c) HCV NS3/4A;^31^ (d) furin;^32^ (e) FRA3 and MPII;^33^ (f) FRA3 and MPII;^34^ and (g) MMPs (1, 2, 3, 7, 8, 9, 10, 11, 12, 13, 14, 15, 16, 17, 19, 20, 24, and 25).^35^ Note that HCV NS3/4A and FRA3 and MPII are both reported in two different manuscripts.

Using the same procedure, the same research group published five further articles examining different proteases. Reported simultaneously, Shiryaev et al. also investigated HCV NS3/4A using a different library design of >2600 8mers from the viral polyprotein.^31^ Their data revealed that there were specific residues important at each site, but they were not always the same; in some cases, they were near the cleavage site (P1/P1′) while others were more distal (up to P6), with a newly observed trend that Asp or Glu was enriched at P6 and P6.

Subsequently, human furin was profiled to better understand its role in development (knockout is lethal in mice) and disease pathologies.^32^ A designed library of 5860 peptides was used following computational steps to identify potential furin cleavage sites in the human proteome: 3260 predicted/known cleavage sites and 2600 non-furin control sequences from other proteases. The results revealed 490 likely sites, far more than the 150 reported in databases at the time. These also highlighted the importance of both short-range (P4–P1) and long-range (P7–P6) interactions for furin specificity and that the previously identified consensus R-X-R/K/X–R↓ motif alone is insufficient for predicting cleavage sites.

Next, the metalloproteases MPII and FRA3, which are virulence factors for *Bacteroides fragilis*, the causative agent of most anaerobic infections in humans, were investigated using a biotinylated 10-mer peptide library of 316 sequences (compiled based on previous results).^33^ Over 80 peptides were substrates for MPII and >230 for FRA3 with different consensus sequence preferences: G­(R/G)­LR↓RX­(R/G) and P­(R/A)­(P/G/S)­L­(R/A/L)↓, respectively. In a subsequent report, MPII and FRA3 were investigated further using a larger 18,583-member designed library.^34^ Different enzyme concentrations were investigated; high concentrations (4 μM) led to greater nonspecific cleavage, while low concentrations (40 nM) diminished the sensitivity but were useful for identifying the most specific substrates. Most data were gained from the intermediate concentration (400 nM) selection, and MPII was shown again to have a preference for Arg/Lys at P1 and Arg at P1′ sites, while FRA3 was less specific with a lower preference for Lys/Arg at P1 and aliphatics (Leu/Val/Met) at P1′.

Lastly, a set of 18 matrix metalloproteinases (MMPs) was investigated using the same 18,583-member 10-mer library as above. Catalytic domain-only constructs were used for screening as it was reasoned that the 10-mer peptides should only occupy the substrate cleft of the MMPs, and not interact with auxiliary domains. To test this a preliminary screen with full-length MMP-2 and MMP-9, as well as catalytic domains only, was performed and found no significant difference. Over 500 substrates with *Z*-score >2.5 (a measure of substrate efficiency based on statistical analysis of read counts in sequencing versus control samples) were identified for all MMPs, except −7 and −19, which were consistent with previous reports and showed that MMPs within the same subfamilies had similar substrate scopes, but were distinct from other subfamilies, although approximately 50% of substrates were cleaved by all MMPs. The data obtained also correlated well with a model previously developed to predict MMP cleavage sites.

Using a different library design with a similar screening approach Iskandar et al. investigated chymotrypsin, a gut endoprotease that cleaves hydrophobic aromatic residues at the carboxyl side of susceptible bonds within proteins.^36^ The activity of chymotrypsin leads to the degradation of peptide drugs, which greatly limits their oral bioavailability, so to facilitate the design of proteolytically stable peptide drugs, mRNA-display was used to determine the identity of resistant peptides. In this study, an N-terminal biotinylated library (N-biotin-l-Phe recoded at the initiator AUG codon using flexizyme) was incubated with chymotrypsin. The library was randomized at six positions using NNW codons (total diversity 3.4 × 10^7^), with the exclusion of Met and Cys during translation allowing Trp to be incorporated at UGU codons through flexizyme-mediated charging (Figure 8). Peptides were incubated with chymotrypsin and then separated using streptavidin; substrates remain in solution, while non-substrates of chymotrypsin retain the *N*-terminal biotin and are immobilized. Recovered peptides were then sequenced.

**Figure 8:**
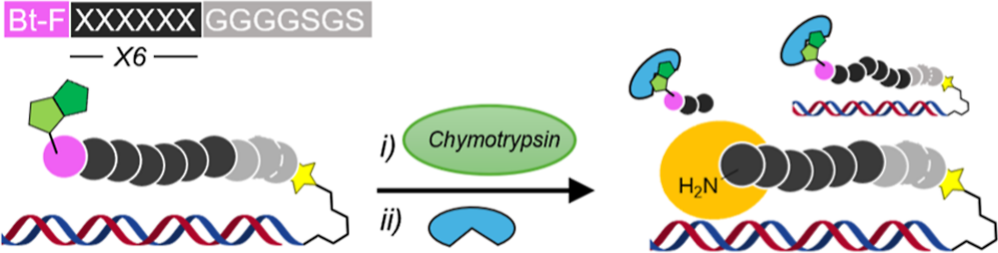
mRNA-display workflow for chymotrypsin substrate identification. N-biotin-l-Phe is genetically encoded at the AUG initiator codon and Trp was recoded to the UGU codon. The library was incubated with chymotrypsin, leading to substrates being cleaved from their biotin handle. Non-substrates retain the biotin handle and are removed via pull-down with streptavidin-coated beads (blue truncated disc).

Selections were carried out in the presence and absence of chymotrypsin. Analysis of the NGS data showed a reduction in the presence of Trp, Phe, Tyr, and Leu residues, consistent with previously reported cleavage sequences in the MEROPS database at the time. They also saw an increase in enrichment of Pro, Glu, and Asp, which suggested that these residues have a stabilizing effect.

Using the NGS data, a simple machine learning-based classifier was trained to predict whether a peptide will be cleaved by chymotrypsin. During the selection, cleaved residues were not recovered or specifically identified; therefore, the purpose of the model was to predict the probability of a peptide being positively identified as stable. The authors validated the results of the computational predictions by synthesizing a set of peptides representing several observed trends and measuring their half-lives in the presence of chymotrypsin. Their computational model showed a good predictive ability with a true positive rate of ∼70% and a true negative rate of ∼80%. Significantly, the classifier could predict that peptides containing either a Phe, Tyr, Leu, or Trp are unstable.

Employing a similar method, Zhu et al. (preprint) determined the specificity of three poorly understood proteases: factor Xa, ADAM17, and streptopain, developing a methodology capable of selecting for both narrow and broad range proteases.^37^ The library comprised 8-mers encoded using nucleotide trimers to eliminate codon bias (see later section for more details), flanked on both sides by a Pro-Gln-Pro “frame” known to be proteolytically stable. The N-terminal constant region also contained a His_6_-tag and the UAA *para*-propargyloxy-l-phenylalanine (pPaF) encoded through suppression of an amber stop codon (UAG); the C-terminal constant region contained a linker sequence. The overall library diversity was 2.56 × 10^10^. Displayed peptides were covalently linked to azide-functionalized agarose beads via a Cu­(I)-catalyzed alkyne–azide cycloaddition (CuAAC) with the pPaF residue and treatment with a protease cleaving the C-terminal region with encoding mRNA/cDNA attached, allowing straightforward recovery (Figure 9).

**Figure 9:**
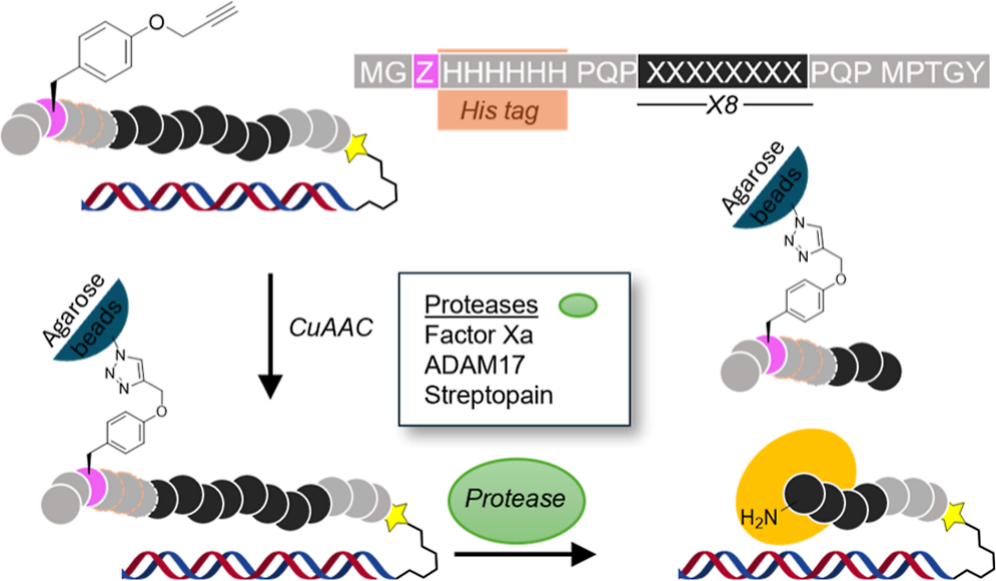
Library design and selection approach developed by Zhu et al.^37^ Amber suppression was used to install an invariant *para*-propargyloxy-l-phenylalanine residue at the position denoted with Z into peptides. Azide-modified agarose beads were then used in CuAAC reaction to immobilize the peptide-mRNA/cDNA conjugates. Incubation of the immobilized library with a protease (Factor Xa, ADAM17 or streptopain) leads to the release of substrate peptides and encoding mRNA/DNA that can be recovered and sequenced. Non-substrates remain immobilized on the beads.

Using this methodology, a G/A-R↓ motif at the P1–P2 positions was identified as a cleavage pattern for the factor Xa protease, consistent with the established site of I-E/D/G-R↓ but no strong preference for positions P3 or P4 was identified. Additionally, a previously unknown Q-R↓ cleavage site was discovered. For ADAM17, screening showed QAV↓ or QRV↓ were the most abundant cleavable motives present in 609 unique sequences. The screening of streptopain showed confirmation with previous studies for the preference of hydrophobic amino acids at position P1; in addition, the preferences for the less well-known positions P3 (Y, I, M, F, and V), and P1′ (F), were also reported. In each case, the two least enriched sequences were also shown to be digestible by their respective protease, which shows the effectiveness and selectivity of this approach. While useful for identifying substrates, the display approach cannot identify the specific site of cleavage within a peptide; this was confirmed in follow-up assays using chemically synthesized peptides and LC–MS analysis. These data were incorporated into a computational model in addition to NGS data to generate specificity motifs.

To understand the implications of UAA incorporation on protelytic activity, Turra et al.^38^ used mRNA-display to probe the activity of methionine amino peptidase (MAP) towards homopropargylglycine (Hpg) a Met surrogate recognized by the methionine tRNA synthase.

Four different libraries were designed, all containing an N-terminal Hpg (encoded at Met, AUG) followed immediately by either Ala or Xaa (random), 4 or 8 randomized amino acids using NNS codons (total diversities AX4: 1.6 × 10^5^; X5: 3.2 × 10^6^; AX8: 2.56 × 10^10^; X9: 5.12 × 10^11^), a C-terminal His-tag and linker region (Figure 10a). The translation step was conducted without Met, which was replaced with Hpg at all positions.

**Figure 10:**
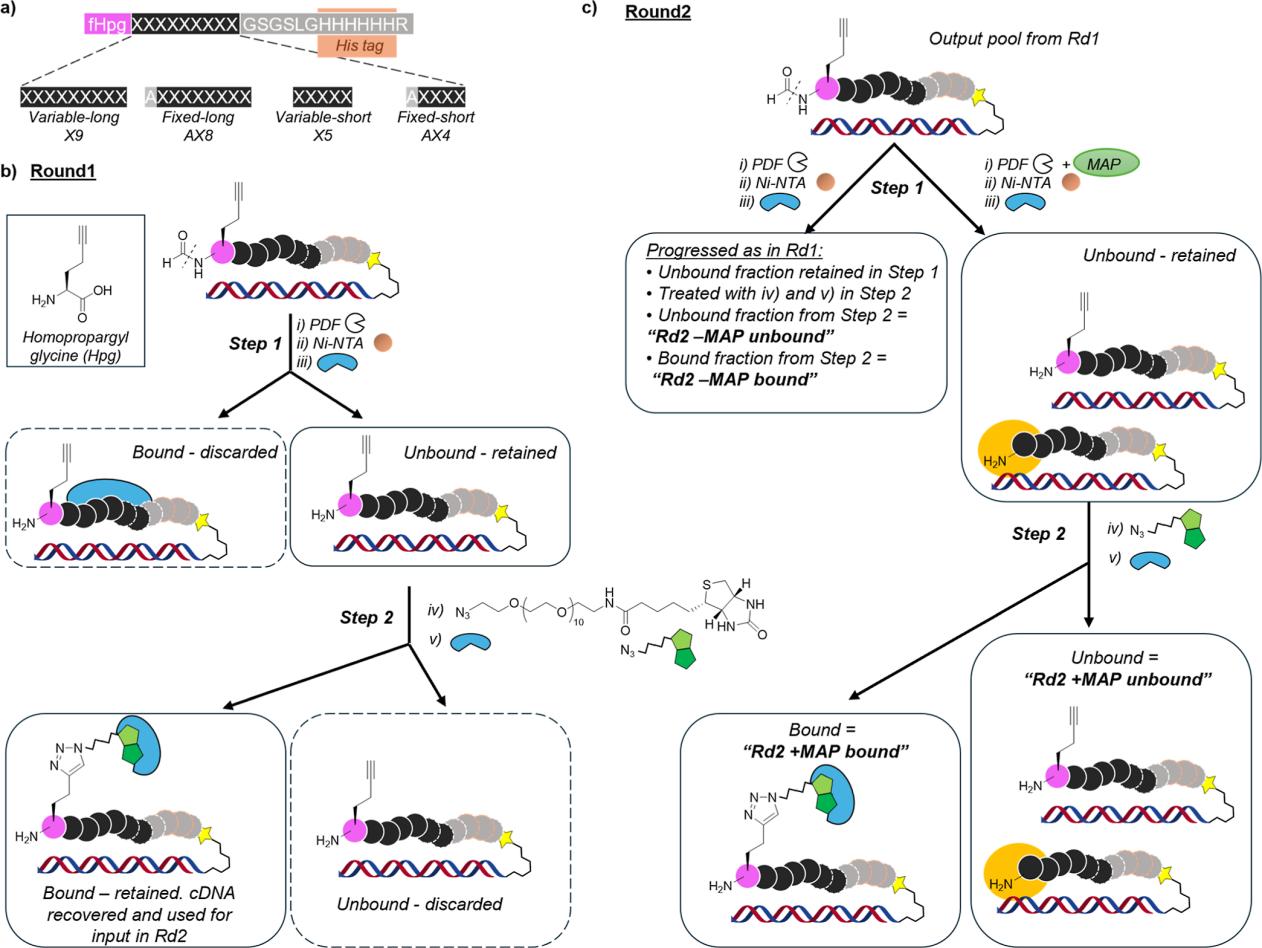
(a) Library designs used by Turra et al. to study MAP activity on the UAA homopropargyl glycine (Hpg, structure show inset in panel b), comprising a conserved Hpg, variable region, spacer, and His-tag. Two rounds of selection were used; (b) round 1–selection without MAP, to preclear the library to leave only sequences that are substrates for peptide deformylase (PDF) (activity of which is required before MAP can function), do not bind streptavidin in their unmodified form and can be “clicked”. Step 1: after incubation with PDF, (i) peptides were purified via His-tag, (ii) then incubated with streptavidin beads (blue truncated disc), (iii) and the unbound fraction retained. Step 2: the library was then “clicked” with biotin-PEG11-azide (iv) and sequences binding to streptavidin beads (v) retained and used as the input for round 2; (c) round 2–identification of MAP substrates. The output library from round 1 was split and one portion treated as previously, with both the bound and unbound fractions from step 2 retained. The other portion was incubated with PDF and MAP during (i), but otherwise the same. Sequences that are substrates for MAP will lose the Hpg residue and this be recovered as part of the unbound fraction in step 2. DNA pools were analyzed by NGS after round 2.

A two-stage process was used to identify MAP substrates for each of the four libraries. In round 1, translated peptides were immobilized on streptavidin beads via a photocleavable biotin contained within the primer for reverse transcription (not shown). While immobilized, reverse translation was performed, followed by treatment with PDF to remove the N-terminal formyl group from the Hpg residue; MAP can only act on N-terminal residues bearing an amino group. Peptide-mRNA/cDNA complexes were released by photocleavage, purified via the C-terminal His-Tag using Ni-NTA agarose, and eluted peptides were incubated again with streptavidin beads, removing any inherently bead-binding sequences. The unbound peptides were then reacted with biotin-PEG_11_-azide before a third incubation with streptavidin beads with immobilized sequences at this point representing correctly translated and ‘clickable’ sequences, from which the cDNA was recovered and amplified as input for round 2 (Figure 10b). Two parallel round 2 selections were then performed for each library, one the same as described above, with the difference that both bound “Rd2 -MAP bound” and unbound “Rd2 -MAP unbound” sequences from the final streptavidin immobilization step were sequenced. For the “+MAP selection” in round 2, libraries were treated with PDF and MAP simultaneously, leading to cleavage of the Hpg from substrate sequences, which would thus be included in the “unbound” pool following the final streptavidin immobilization. As for the “-MAP selection”, both bound “Rd2 + MAP bound” and unbound “Rd2 + MAP unbound” sequences from the final streptavidin immobilization step were sequenced.

Combining information from all sequenced pools revealed that the major determinant for substrate fitness was the P1′ position, with a strong preference for amino acids with small side chains (e.g., Gly, Ala, Ser, and Pro), while charged and bulky residues (e.g., Asp, Lys, Tyr, and Trp) were poorly accepted. These groupings were consistent with previous findings, though the average cleavage efficiency varied from 85%–20%, whereas it was anticipated that some substrates would be quantitatively cleaved and others would not be cleaved at all (i.e., 100% or 0%). Various reasons for this are suggested, including that the values are averages, so there may be specific sequences, which are 100% or 0%, depending on the downstream residues. To investigate downstream residues further, they examined substrates with residues at the P1′ position that gave ∼50% cleavage, revealing some effects at the P2′ (e.g., Trp favoring cleavage and Pro or Glu disfavoring it), P3′ and P4′ positions, but these diminished with increased distance. Cys-containing peptides were strongly disfavored, contrasting with previous literature reports.

Overall, substrate profiling of proteases by mRNA-display is a relatively straightforward undertaking; an *N*-terminal affinity tag is all that is needed to separate substrates from non-substrates and is perhaps why so many proteases have been studied in this way. Crucially, though mRNA-display cannot identify the cleavage site directly, and thus follow-up work with individual peptides is required to get the full picture.

### Transglutaminases

The transglutaminases (TGs) are a class of enzymes that post-translationally modify proteins by catalyzing the cross-linking of the ε-amino group of lysine residues to the carboxamide of a glutamine residue. The TGs are involved in cell differentiation, signal transduction, and cancer cell metastasis. To understand how TGs activate these physiological processes, an understanding of the peptide substrate specificity of these enzymes could be valuable and multiple groups have investigated this using display technologies.

Previously, Keresztessy et al.^49^ and then later Sugimura et al.^50^ conducted phage display campaigns against transglutaminase-2 (TG2) and microbial-transglutaminase (mTG), respectively. For TG2, a consensus sequence of pQX­(P,T,S)­l (where p and l indicate polar and aliphatic amino acids, respectively) was identified, while for mTG, the most enriched motif was LxRQRY. However, the residue characterizations were not conclusive, and subsite specificities remained elusive. Since then, several further reports have used mRNA-display to characterize TG substrates.

A report from Lee et al. built on this work by using mRNA-display to select against *Streptomyces mobaraensis* transglutaminase (STG).^39^ The authors comment on the significantly larger library size (10^9^ versus 10^13^) achieved in their work compared to the phage-display campaigns. The library was designed to encode a randomized region of ten residues, encoded by NNS codons (total diversity 1 × 10^13^). A capture style assay was used, where hexa-lysine (K_6_) beads, acting as the amine donor, captured reactive substrates that were cross-linked after incubation with STG (Figure 11).

**Figure 11:**
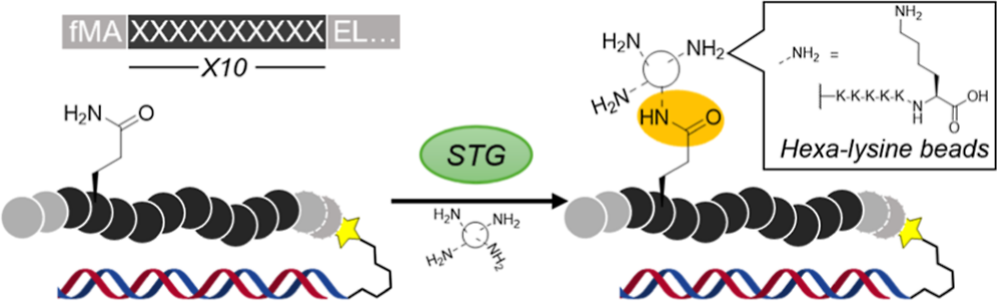
Transglutaminase capture assay from Lee et al.^39^ using functionalized hexa-lysine beads. Upon incubation with STG, reactive glutamine substrates become cross-linked to the beads, allowing recovery and encoding mRNA to be sequenced.

After six rounds of display, 105 were identified and aligned by fixing a potentially reactive Q-residue, highlighting Pro at +1 to be the most frequently observed amino acid and Arg to appear significantly at position −3. Partial consensus sequences were observed with LQQ and TQP being repeated eight and seven times, respectively. Follow up work showed tripeptide substrates were inactive with STG and pentapeptides were the minimum active substrate length. On the basis of the sequence alignment data, the authors designed a pentapeptide RLQQP (TQ1) based on the sequence ASMERLQQPN, identified from the selection, which was concluded that TQ1 was a more specific substrate for STG than TG2.

In a later study, Damnjanović et al. (2022) used mRNA-display to identify substrates for TG2.^40^ The mRNA-display library was designed based on the previously known substrate of TG2, termed T26.^50^ In their library, the T26 peptide was randomized at positions −1, +1, +2, and +3 relative to the reactive glutamine residue (Figure 12a). Randomization was introduced by NNK codons, generating a 1.6 × 10^5^-member library. During incubation with TG2, pentylamine-biotin (PAB) was employed to act as the amine donor, which results in the biotinylation of reactive glutamine substrates. The cross-linked substrates can then be separated via pull-down with streptavidin-coated magnetic beads (Figure 12d).

**Figure 12:**
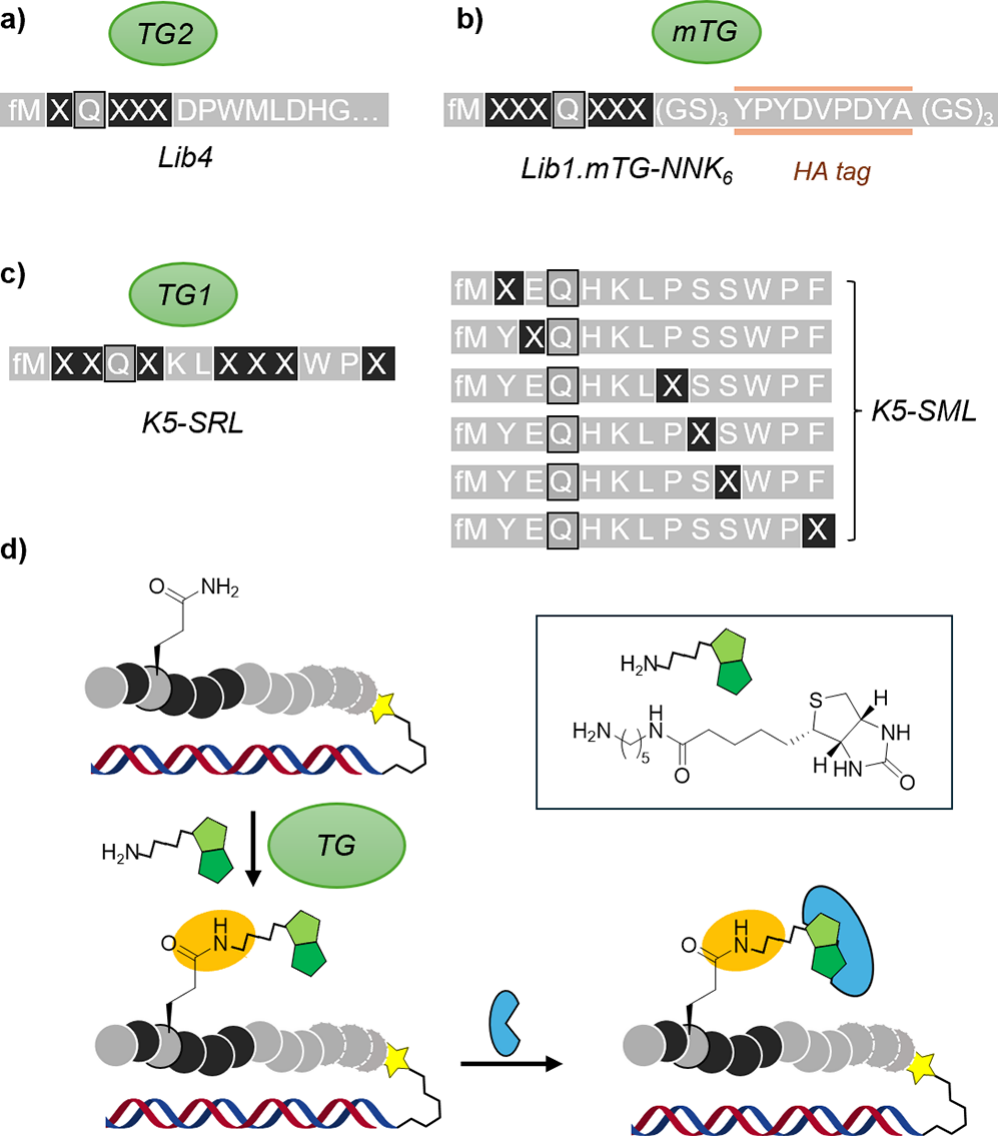
Library designs used to investigate the transglutaminases (a) TG2;^40^ (b) mTG;^41^ and (c) TG1.^42^ All contain a conserved invariant Gln (gray with black outline) and various combinations of invariant and varied residues; (d) design of capture assay used for all three enzymes using pentylamine biotin (inset), which can intercept reactive glutamines thereby biotinylating substrates that can be recovered via pull down with streptavidin-coated magnetic beads (blue truncated disc). The library design used for TG2 in part a is used as an example.

This work identified a strong preference of TG2 for peptides with a Gln at position −1, in concordance with previous reports identifying a substrate Gln–Gln motif. A preference for Ile or Val at position +3 was also identified. The authors highlight some differences between their results and those previously achieved through phage display. The current study did not find a preference for Pro at position +2. In addition, the T26 peptide sequence was found to rank >2000th with an enrichment factor of 2 in their data set, compared to the top hit with an enrichment factor of 7 (fMQQVCIDPWMLDH).

In 2023, Bowler et al. sought to exploit the activity of mTG to establish a novel strategy of macrocycle generation in mRNA-display libraries, to then identify potent cyclic peptide inhibitors.^41^ Having established that mTG is active with mRNA-display reactive Gln substrates, they used PAB to capture substrates during mTG treatment (Figure 12d) using a peptide library comprising an initiator Met, 3 random residues (encoded by NNK codons) flanking an invariant Gln, Gly-Ser spacer and HA-tag. As reported for TG2, NGS analysis revealed two key trends: (1) aromatic residues were enriched on both sides of the reactive Gln; (2) amino acids affecting peptide flexibility (Gly and Pro) were under-enriched at all sites. Overall, the NGS data revealed that no single sequence motif was enriched (<0.03% sequence convergence); therefore, mTG shows minimal substrate bias, a beneficial characteristic for cyclic peptide library generation.

They next evaluated mTG compatibility and bias with mRNA-display substrates during the Gln to Lys cross-linking step. They designed and screened two libraries, both with N-terminal *N*-biotin-l-Phe incorporated at the start codon by flexizyme reprogramming, followed by four or six randomized positions encoded with NNK codons flanked by Gln and Lys (termed NNK_4_ and NNK_6_ with diversities of 1.6 × 10^5^ and 6.4 × 10^7^, respectively). After treatment of the libraries with mTG, trypsin protease was employed to cleave at the unmodified Lys in the uncyclized substrates. This removes the biotin tag from the genetic material and prevents sequence enrichment of non-substrates. Analysis of the NGS data revealed similar trends to their previous assay, with high sequence diversity observed and Pro being the most under-enriched amino acid. A notable trend observed was the enrichment of Gln residues closer to the reactive C-terminal Lys, which could suggest that macrocycle ring sizing may have a stronger impact on cyclization than the sequence context. Overall, authors conclude that mTG does not introduce significant substrate bias into a cyclic peptide inhibitor selection, and they proceed to use it in this context with the identification of cyclic peptide binders against the calcium and integrin-binding protein (CIB1), and the immune checkpoint protein B7–H3. Their cyclic peptide selection identified high-affinity, low-nanomolar inhibitors of these targets, and these macrocycles showed a high sequence diversity.

In further work from the Nakano group, Munaweera et al.^42,40^ investigated the substrate preferences of transglutaminase-1 (TG1). TG1 is of known importance to skin development, and aberrant expression of this enzyme is suspected to be linked to Alzheimer’s.

The library design was based on the previously reported K5 peptide YEQHKLPSSWPF,^50^ which showed high reactivity and selectivity for TG1. One library, K5-SRL, varied seven positions within 5 simultaneously with NNK codons XXQXKLXXXWPX (total diversity: 1.3 × 10^9^ peptides). The other library used, K5-SML, contained single-point mutants in which one of the six position within the peptide xxQHKLxxxWPx was randomized, giving 19 different variants at each position with a total K5-SML diversity of 115 (Figure 12c). As previously mentioned, PAB was used to capture and separate the cross-linked substrates fused to their encoding mRNA.

The NGS data from library selections were analyzed to reveal Pro at position +4, Glu at −1, and Phe at +9 to be important for sequence enrichment. Interestingly, selection from the K5-SML library identified a more reactive sequence, pep 1–1: *A*EQHKLPS*K*WPF (changes from parent K5 peptide italicised), which also had the greatest specificity for TG1 over TG2 and TG3. The results of these studies were the identification of highly active peptides that could be used as probes for further study into these enzymes. In particular, the substrate preference information could enable the identification of human protein targets of the TGs.

### RiPPs

Ribosomally synthesized, post-translationally modified peptides (RiPPs) are a class of natural products comprising extensively modified peptides that arise through multiple PTMs to a ribosomally synthesized precursor. Modified sequences consist of a recognition sequence N- or C-terminal to the modified region (termed leader and follower sequences respectively), which decouple substrate recognition from substrate modification and enable diversity in the modified region. Many RiPPs have interesting biological activities, and multiple reports have emerged in recent years investigating the substrate scope of the modifying enzymes.

In 2020, Fleming et al. investigated the pantocin biosynthesis enzyme (PaaA), capable of forming the pantocin indolizidinone core from three amino acids Glu16-Glu17-Asn18 in the wild-type precursor peptide (Figure 13a).^43^ Initially, a saturation mutagenesis single variant library (smSVL) was constructed, where each residue in the precursor peptide (i.e., leader, core, and follower regions) was mutated to all other amino acids, creating a library of ∼550 single mutants (Figure 13b). The UAA *N*-biotin-l-Phe was incorporated as a reprogrammed initiator using flexizyme for all peptides, which also had a conserved C-terminal region, including an HA-tag. mRNA-display libraries were exposed to PaaA and purified via HA-tag before a post-modification step using GluC to cleave the peptide backbone C-terminally to Glu residues, thus separating any unmodified core sequences from their encoding mRNA. Capture was then performed using streptavidin (Figure 13c).

**Figure 13:**
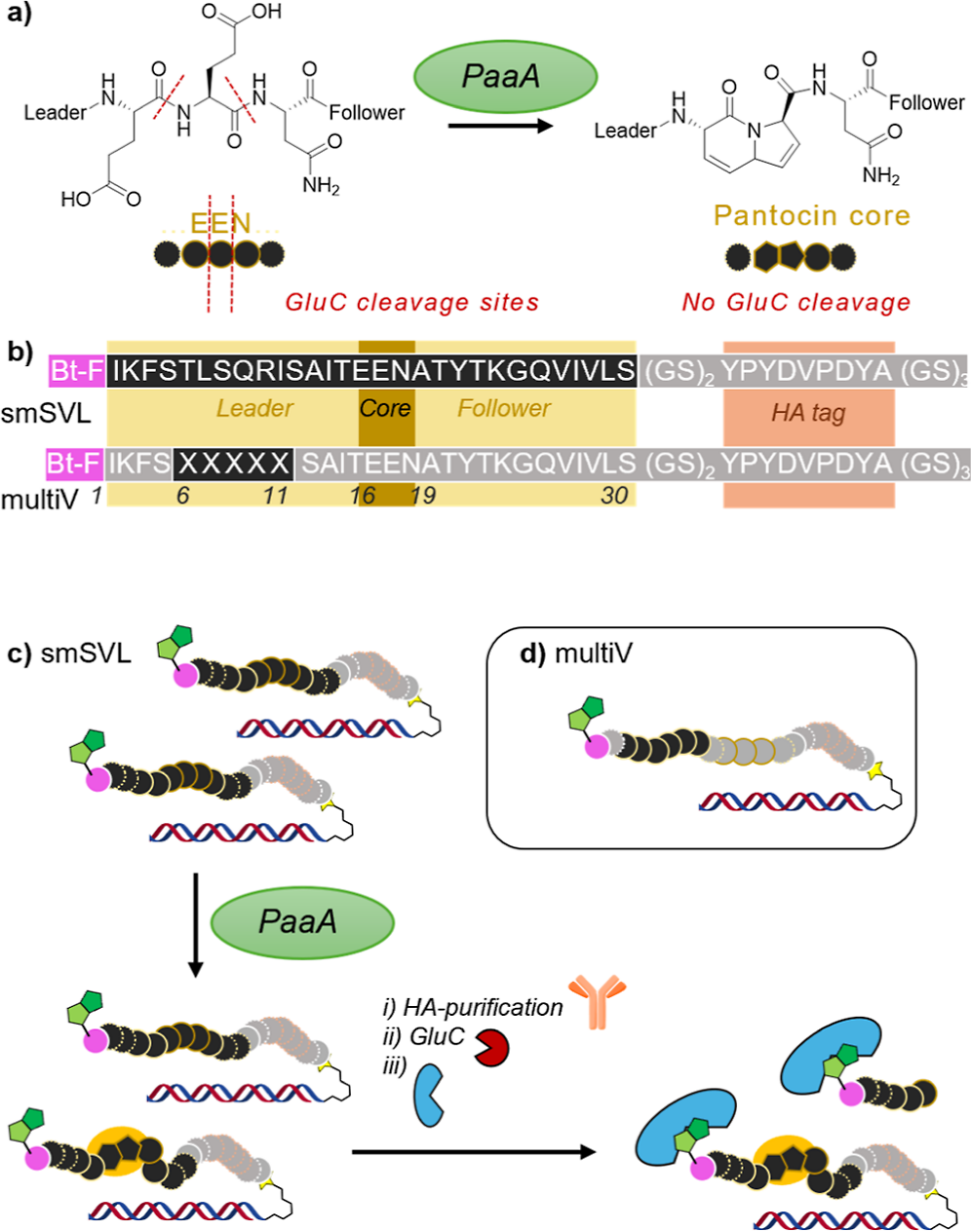
Investigation of pantocin A synthase PaaA. (a) The precursor peptide contains Glu residues that can be cleaved by the protease GluC. Generation of the pantocin core by the action of PaaA removes potential GluC cleavage sites; (b) library designs used for mRNA-display. The smSVL (top) consisted of point mutants at each position with the leader (beige outline), core (brown outline), or follower (beige outline) sequence. The multiV library (bottom) contained saturating mutations through NNK codons at positions 6–11 (part of the leader sequence) with the rest of the sequence invariant. Both libraries were initiated with a reprogrammed initiator of *N*-biotin-l-Phe (pink circle) and had a conserved C-terminal region including HA-tag (peach outline); (c) PaaA substrates are discriminated by their resistance to the protease GluC (red truncated disk). All peptides have N-terminal biotin and internal Glu residues; upon action of PaaA, Glu residues in substrates are converted into the indolizidinone core, rendering those peptides resistant to GluC cleavage. Following incubation with PaaA, all peptides are recovered via the HA-tag, treated with GluC and then with streptavidin beads (blue truncated disk) to discriminate between substrates (retained in solution) and non-substrates (captured on beads); (d) representation of the input multiV library, shown in detail in (b), which was selected in the same way as depicted in (c).

Following a single round of selection with various PaaA concentrations, enrichment scores were calculated for each amino acid at each position. Glu-containing peptides were heavily depleted, as would be expected by the selection procedure; regardless of pantocin core formation, these substrates would be cleaved by GluC and thus not recovered. Otherwise, these data revealed that Phe3, Leu6, Arg10, and Ile11 within the leader sequence were highly sensitive to change; Pro within the core region was not tolerated, but Glu17 and Asn18 were reasonably tolerant to mutation otherwise, while the accepted mutations to Glu16 were extremely limited. The authors note that modifications to Glu16 or Glu17 in the core region would preclude mature indolizidinone formation as it requires cyclization and dehydration of one residue followed by decarboxylation of the other, though the order was not known (i.e., 16 then 17 or 17 then 16). From their data, the authors inferred that Glu16 was likely the first residue (undergoing cyclization and dehydration) as it was the most intolerant to mutation; this was confirmed through follow-up experiments with chemically synthesized, isotopically labeled peptides.

To further investigate the important residues within the leader sequence, a second library was designed with combinations of saturating mutations at positions Thr6-Ile11 (through NNK codons) with the rest of the sequence invariant (total diversity 6.4 × 10^7^) (Figure 13b and d). Using the same selection procedure on this library reinforced the previous findings: Arg10 was essential, while only aliphatic hydrophobics at positions 7 and 11 were tolerated (Leu7 → Val and Ile11 → Val/Leu). Positions Thr6, Ser8, and Gln9 were highly tolerant but showed some preference for Arg or Ser.

Another class of RiPPs, thiopeptides, are characterized by multiple side chain to backbone cyclizations forming (methyl)­oxazoles or thiazoles, as well as, uniquely, formation of a pyridine ring through peptide macrocyclization involving the N-terminus. Expanding on work to use RiPP enzymes to generate large screening libraries, Vinogradov et al. sought to understand the substrate tolerance of the biosynthesis enzymes (LazBCDEF) for lactazole A, an unusual thiopeptide from *Streptomyces lactacystinaeus* (Figure 14a).^44^ In keeping with convention in the RiPP field and the cited works, we use LazBCDEF to represent the combination of lactazole biosynthesis enzymes used here; i.e., LazBCDEF is a mixture of 5 enzymes: LazB, LazC, LazD, LazE, and LazF.

**Figure 14:**
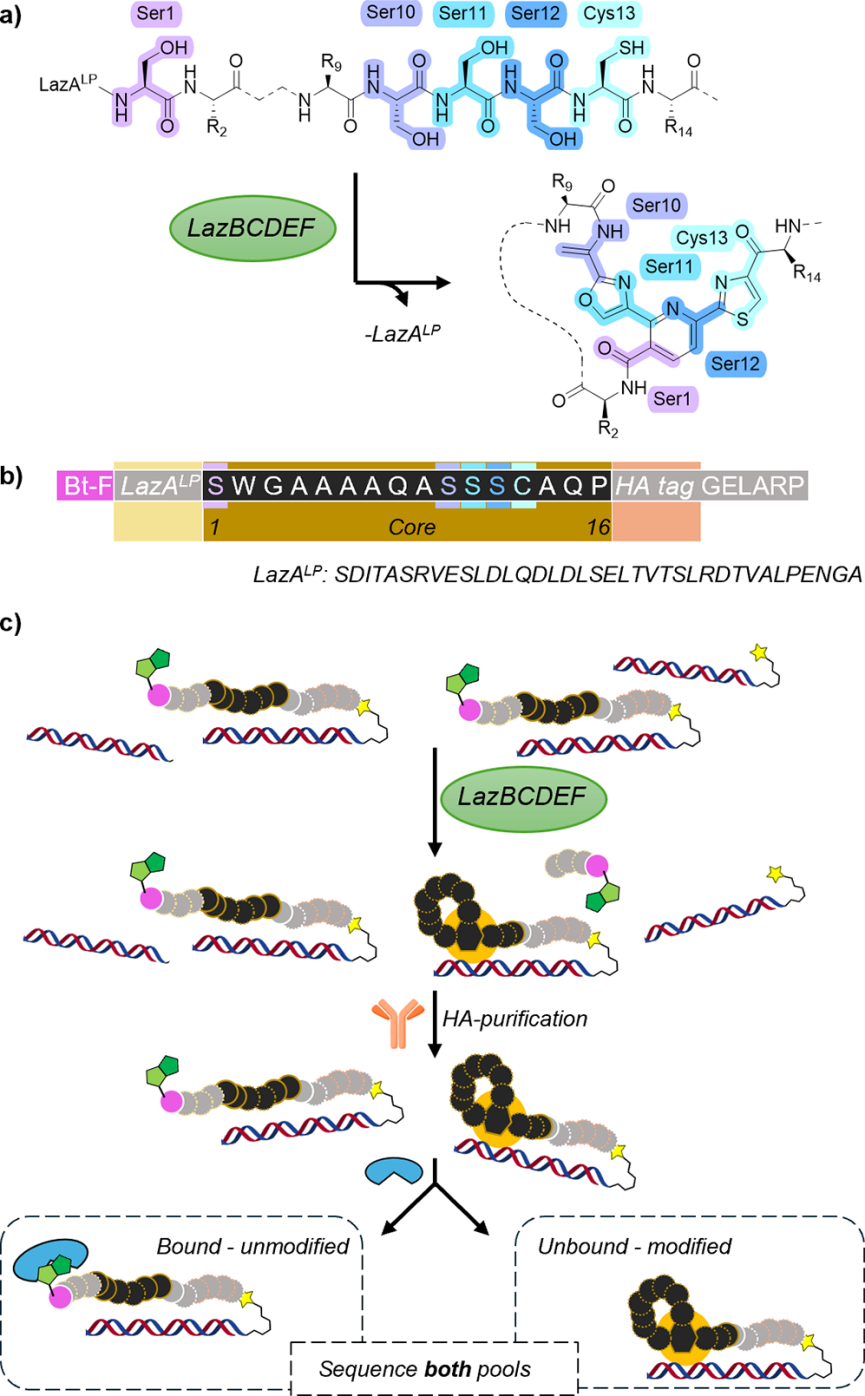
(a) Modification of key residues within the core of Laz A forming a dehydroamino acid, oxazole, thiazole, and pyridine ring from Ser and Cys residues, which are highlighted to trace the originating amino acids contained within the modifications; (b) a saturating mutagenesis single variant library design was used with each residue in turn mutated to all other 19 nAAs with the rest of the sequence invariant. All peptides contain an *N*-terminal biotin-l-Phe, LazA leader peptide, smSVL variable region based on LazA^min^, and HA-tag. Modified residues are highlighted with the same colors as in (a). Sequences of the leader peptide and HA-tag are abbreviated for clarity; (c) following exposure of the translated library to LazBCDEF, all peptides are recovered via the *C*-terminal HA-tag, then non-substrates removed through incubation with streptavidin beads (truncated blue disk). Fully modified substrate peptides contain a pyridine ring, which results in loss of the *N*-terminal leader peptide containing the biotin moiety and thus they cannot bind streptavidin. HA-tag = YPYDVPDYA.

In a similar approach, a smSVL library of 304 sequences covering just the core region was used, based on a previously identified minimal lactazole A sequence, LazA^min^, with Ala mutations at four positions compared to wtLazA (Figure 14b).^51^ The UAA *N*-biotin-l-Phe was incorporated as a reprogrammed initiator for all peptides, which also had the LazA leader peptide at the N-terminus and an HA-tag at the C-terminus. mRNA-display libraries were exposed to LazBCDEF and purified via HA-tag; the authors note this is an essential step to remove frame-shift mutants, Pu-mRNA without peptide attached and unligated mRNAs (i.e., lacking the Pu-linker). Capture with streptavidin was then used to discriminate between mature and partially modified thiopeptides; during pyridine formation, the *N*-terminal leader sequence is cleaved as the backbone of the first amino acid of the core forms part of the pyridine ring (Figure 14a). Hence, mature thiopeptides will lack the N-terminal biotin-l-Phe, not bind to streptavidin, and remain in solution, while partially modified peptides will retain their streptavidin binding (Figure 14c).

A key advance shown here was the recovery and analysis of both the unmodified and the modified sequences, with the HA-tag purification ensuring final pools comprise only substrates and non-substrates. Previous studies had used enrichment scores by comparing modified sequences vs input, but with these two well-defined pools, *Y* scores for each peptide were calculated; defined as the ratio of frequencies in modified and unmodified populations. *Y* scores gave a larger signal response and were more accurate. The single-point mutation data were largely consistent with previous findings; residues 4–7 were highly mutable (all of these were already mutated to Ala from wtLazA in the LazA^min^ sequence used here) with approximately one-third of the library comprising substrates of higher fitness than the LazA^min^ scaffold. Overall substrate-level cooperation between the biosynthetic enzymes was shown to maintain the integrity of lactazole assembly to converge on a single product.

During lactazole A biosynthesis, LazB glutamylates serine (or threonine) residues, enabling subsequent elimination to form dehydroamino acids (cf. Ser10, Figure 14a) by a separate, nonspecific dehydratase (LazF). To investigate the specificity of LazB (Figure 15a), Vinogradov et al.^45^ further developed the previous approach of capturing both substrate and non-substrate sequences and using them through multiple parallel rounds of mRNA-display termed “selection” and “anti-selection”. The “selection” enriched for substrate (modified) sequences each round, while the anti-selection enriched for non-substrate (unmodified) sequences each round (Figure 15d).

**Figure 15:**
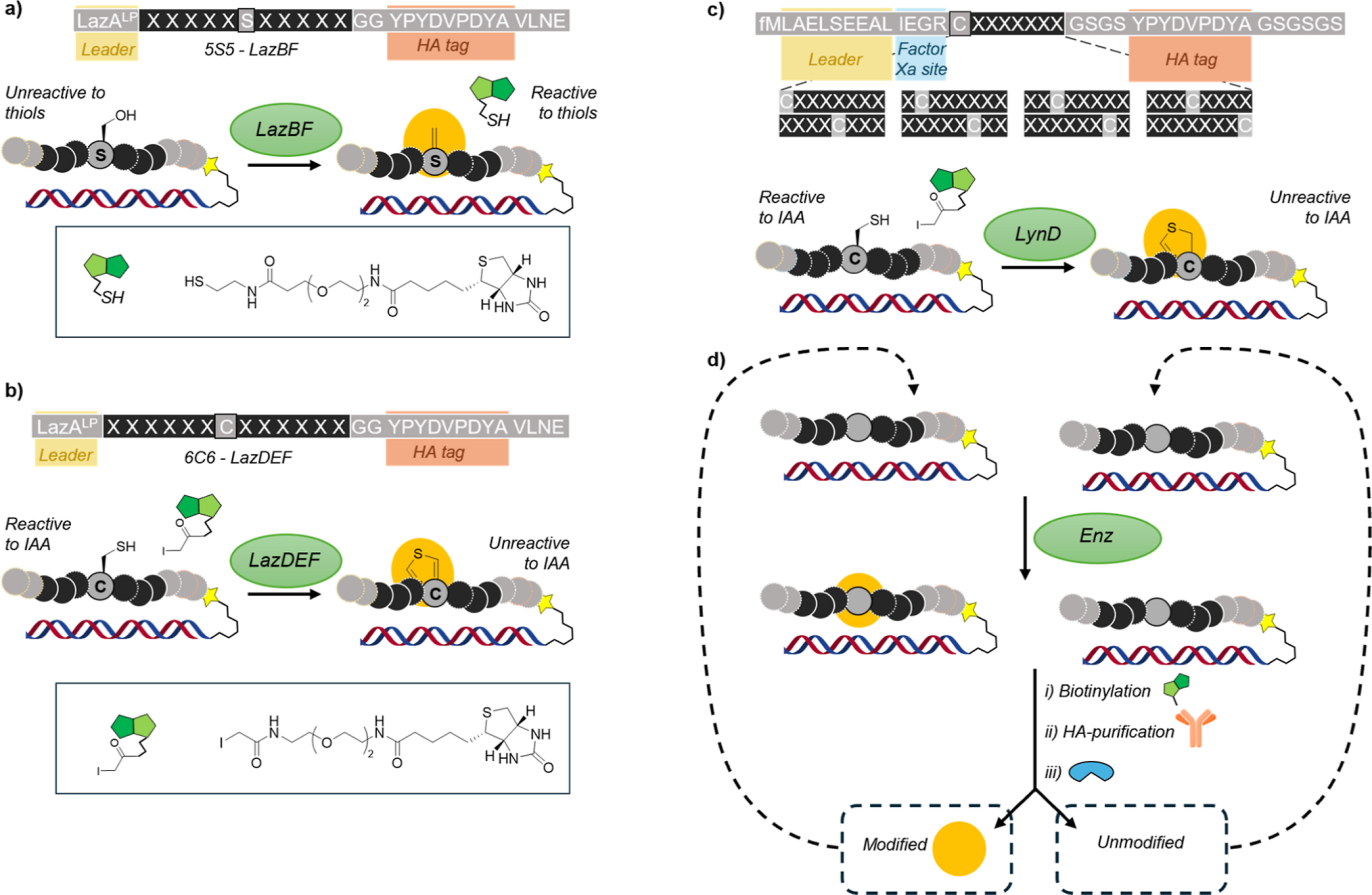
Library designs, target enzymatic modification, and post-enzymatic modification steps for (a) LazBF;^45^ (b) LazDEF;^45^ and (c) LynD.^46,47^ (d) The shared workflow for iterative enrichment of substrates and non-substrates via post-enzymatic discrimination with biotinylation reagents. In (a), substrates are reactive to the biotinylated thiol probe, whereas in (b,c), substrates are unreactive to the biotinylated iodoacetamide probe.

The starting library comprised an N-terminal LazA leader sequence, a conserved Ser flanked by 5 variable residues (dubbed 5S5) and a C-terminal HA-tag (Figure 15a): theoretical diversity 1 × 10^13^. Following reaction with LazBF, Ser residue(s) in substrates would be converted to dehydroalanine (Dha) and thus be reactive to a biotinylated thiol probe, which was used in a post-enzymatic modification step prior to HA-tag purification and capture of substrate peptides using streptavidin beads. Six rounds of mRNA-display selection were performed, and *Y*-values were determined, showing high enrichment of aromatic residues (particularly Trp) at all positions, Ser throughout (in addition to the invariant central Ser), Leu at position 5, and depletion of sequences containing Cys, Asp, Glu, and Lys (though not Arg). Further in-depth bioinformatic analysis was conducted, including deep-learning approaches to determine the contributions from individual residues to substrate fitness and inter-residue dependencies (also termed epistasis or subsite cooperativity), enabling extrapolation of predictions beyond the peptides contained within the NGS data set. A His-Pro-Ser-Arg-Trp motif around the invariant Ser (underlined) was identified, which made particularly efficient substrates. Overall, the substrate landscape was shown to be highly complex and unintuitive, with second-order epistatic effects between residues dominating the fitness landscape.

Within the same paper, the enzymes generating azoles from Ser, Thr, or Cys residues in lactazole A biosynthesis, LazDEF, were also investigated by the same approach. An analogous library was prepared, replacing the 5S5 region with a conserved Cys flanked by 6 variable residues (dubbed 6C6). The theoretical diversity of this library is 4.1 × 10^15^ but based upon the procedure described for library preparation, we estimate the maximum achieved diversity was 7.5 × 10^12^ (250 nM of primer in 50 μL PCR reaction corresponds to 12.5 pmoles or 7.5 × 10^12^ molecules). Upon exposure to LazDEF, Cys residue(s) in substrates would be converted to azoles and thus be unreactive to a biotinylated iodoacetamide (Bt-IAA) probe (Figure 15b), which was used in the post-enzymatic modification step, enabling discrimination of substrates based on binding to streptavidin in the opposite format to for LazBF, i.e., unmodified substrates are captured on the beads while modified sequences remain in solution. Five rounds of mRNA-display selection and similar bioinformatic analysis were performed. While more straightforward than for LazBF, simple position-wise contributions from amino acids alone could not account for the observed results. Small residues at positions 6 and 8 (flanking the invariant modified Cys7) were highly preferred, and acidic residues (Glu/Asp) were disfavored generally, but efficiently modified peptides with Asp8 were also observed, accounted for by second-order interactions with other residues. Cys was disfavored at all positions, but this may be an artifact of the method used; for sequences with multiple Cys, if LazDEF are unable to convert all of them to thiazoles, then they would be reactive to Bt-IAA and thus included in the anti-selection (i.e., false negatives). The large contribution of epistasis (second-order effects) to substrate fitness would be very difficult to unravel without the depth of sequences characterized in this study, highlighting one of the major strengths of mRNA-display.

Using an analogous approach, the substrate preferences of the azoline-forming enzyme (cyclodehydratase) LynD involved in aestuaramide biosynthesis were investigated by Steude et al.^46^ Lyn D is homologous to LazDE (i.e., LazD + LazE, the split cyclodehydratase from lactazole biosynthesis, which requires both partners for activity), and in this study, no dedicated dehydrogenase was included (c.f. LazF used previously), so the products are expected to be thiazolines rather than the aromatic thiazoles from previous work.^45^

The starting library comprised the Lyn D leader peptide, a Factor Xa cleave site (though this was not utilized in this report), variable region, and C-terminal HA-tag (Figure 15c). The overall library was formed of 8 sublibraries in which the variable region comprised 7 variable residues (achieved through NNK codons) with an invariant Cys at each position, i.e., CX7, XCX6, X2CX5,..., X7C. The invariant Cys was encoded by UGU to distinguish from Cys codons generated from NNK codons at the randomized positions, which would be UGC. The overall library diversity was 1.0 × 10^10^ (1.3 × 10^9^ per sublibrary).

The library was incubated with LynD, then Bt-IAA, purified via HA-tag, and captured on streptavidin beads (Figure 15d). As previously, unmodified substrates (anti-selection) will be biotinylated and thus captured on the beads, while thiazoline-containing peptides will remain in solution. Some shortcomings to this approach are highlighted, including that peptides with multiple Cys residues may be only partially modified by LynD (and thus reactive to Bt-IAA and recovered as non-substrates) leading to false negatives, and the potential for IAA to react with residues other than Cys, again leading to false negatives. Nevertheless, after 3 rounds of selection (and anti-selection), the pools were sequenced, and the resulting data were used to calculate *Y*-scores and train a deep-learning model to accurately predict the acceptability of a substrate by LynD.

Cys was highly disfavored at positions 1–4, suggesting that LynD is unable to cyclodehydrate these positions specifically, which the authors note could be due to insufficient distance from the leader peptide; the closest modified Cys in the natural substrate is at the equivalent position to position 6 in the variable region of the library. Unlike LazDEF, Cys was favored at some positions, indicating that LynD can generate multiple thiazolines. Lys was also highly disfavored and could potentially react with IAA, but this was not observed in the previous study^45^ and was concluded to likely be a characteristic of the enzyme; other charged residues (Asp, Glu, and Arg) were also generally disfavored.

From position-wise analysis hydrophobic AAs were enriched in general, residues C-terminal to the invariant Cys had less impact on substrate fitness than N-terminal positions and charged residues near the modified Cys were highly disfavored. Using a more advanced deep-learning model showed some epistasis, particularly between the positions adjacent to the modified Cys but in general the contribution to substrate fitness from epistasis was minimal, unlike LazDEF previously.

In later work, Vinogradov et al.^47^ focused on identifying the substrate specificity of the 5-deazaflavin (F_420_H_2_)-dependent dehydroamino acid reductase, LanJ_C_, using mRNA-display. Dehydroamino acid reductases (dhAARs) generate d-Ala from Dha, so this is one of the few known examples where the enzyme investigated acts upon an already modified amino acid; pre-enzyme modification steps were essential, and these requirements were reflected in the library design.

The library included an *N*-biotin-l-Phe as reprogrammed initiator, constant region including a TEV protease site, variable region of various lengths comprising amino acids encoded with NNK codons flanking an invariant methyl selenocysteine (Sec­(Me), denoted U*) UAA incorporated through recoding a methionine (AUG) codon and a C-terminal constant region including a HA-tag (Figure 16a). Met and Cys were excluded from the translation reaction to avoid oxidative damage in subsequent steps, also generating vacant codons for reprogramming to incorporate *N*-methyl-l-Alanine at the Cys UGC codon. UAAs were incorporated via flexizyme charging. Various lengths of variable amino acids were included around the Sec­(Me), X8U*X8, X5U*X10, X10U*X5, and X6U*X8, with a combined theoretical diversity of 6.9 × 10^20^ (estimated maximum diversity used was 3.6 × 10^12^; the number of mRNA molecules used as input for the translation step).

**Figure 16:**
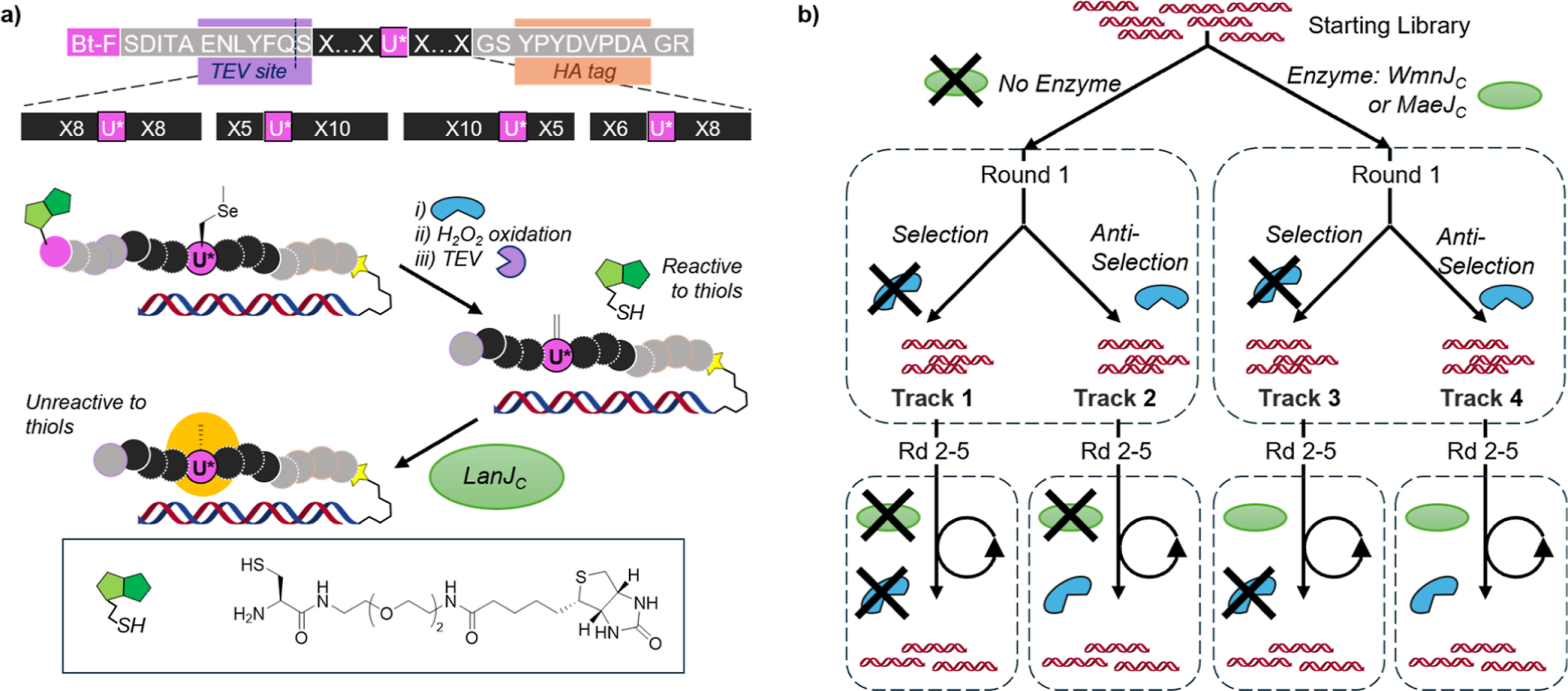
Four-track mRNA-display approach used to identify substrates of LanJ_C_ enzymes, MaeJ_C_ and WmnJ_C_.^47^ (a) Library design enzymatic, pre-enzymatic modification to generate Dha from Sec­(Me), enzymatic modification to generate d-Ala and post-modification step with Cys-PEG_2_-biotin; substrates are unreactive, while non-substrates are biotinylated. (b) The four tracks used to assess background reactions and create better data for computational models.

A pre-enzymatic modification step was used to generate the Dha residues required for LanJ_C_s. The library was immobilized on streptavidin beads via the N-terminal biotin and treated with hydrogen peroxide to induce oxidative elimination of Sec­(Me) residues followed by TEV cleavage to release the peptides (Figure 16a). The immobilization and cleavage were important steps for the removal of translation components and residual peroxide, which otherwise would inhibit the down stream enzymatic reaction. Subsequent steps were as previously: treatment with LanJ_C_ enzymes will reduce the Dha in substrate sequences to d-Ala, rendering them unreactive to thiols. A post-enzymatic modification step involving a reaction with Cys-PEG_2_-biotin allows for the discrimination of substrate and non-substrate sequences after HA-tag purification and streptavidin bead capture. A consequence of the previously established workflow is that products from incomplete modification at any steps will be recovered as part of the selection, e.g., unoxidized Sec­(Me)-containing peptides, off-target TEV cleavage products, or unligated Dha peptides. Thus, a “4-track” workflow was implemented comprising selection and anti-selection experiments, as previously (tracks 3 and 4). In this case, the selection (track 3) of modified substrates should contain d-Ala, not react with Cys-PEG_2_-biotin and thus remain in solution during the final capture step, while anti-selection (track 4) of unreduced Dha-containing sequences will be biotinylated and captured on the beads (Figure 16b). Additional control selection and anti-selection tracks were included performed identically but without the addition of a LanJ_C_ enzyme (tracks 1 and 2, respectively). The combined data from all four tracks were used to train a deep-learning model to identify and remove false positives from the enzyme selections. Examination of tracks 1 and 2 data showed strong enrichment of 3 motifs: Sec­(Me)-Pro, Sec­(Me)-^Me^Ala, and Sec­(Me)-Xaa-Xaa-His. Follow-up work revealed that the first two are highly resistant to the oxidative elimination reaction, so they do not form Dha; consequently, they are not captured by the biotin-thiol modification step and thus recovered within the soluble fraction as substrates. The His residue in the third motif was suspected to undergo an intramolecular reaction with Dha (as indicated by mass-spec data), again rendering it unreactive to thiols.

From this deep-learning analysis, the authors found that in the positive selection against WmnJ_C_, 93.6% of the identified sequences were identified as noise and were therefore discarded. This correlated with the observed round-to-round enrichment for WmnJ_C_, which was similar to the control selection (track 1). Despite the very high background recovery rate, deep-learning analysis could still be performed on the remaining data, showing WmnJ_C_ to have a relatively limited substrate scope (perhaps contributing to the observed high background recovery in the selection), but nevertheless, that any information could be extracted at all is a startling result, likely impossible without the denoising step.

In contrast, MaeJ_C_ was shown to be highly promiscuous and was predicted to modify >35% of all Dha peptides. Further detailed investigation showed that the fitness profiles of both MaeJ_C_ and WmnJ_C_ could be determined by a sum of the amino acid fitness values, with minimal second-order effects. Substrate fitness is driven primarily by hydrophobic interactions in the tetrapeptide region (−2 to +2) surrounding the reactive Dha residue, but more distal hydrophobic interactions can also be important. MaeJ_C_ was shown to have a preference for Ala, Phe, Ile, and Val at a position −1 relative to the Dha. In a final experiment, the authors used the information gained about MaeJ_C_ and previous findings on the enzymes involved in lactazole A biosynthesis to generate a hybrid cyclic peptide structure containing a d-Ala as well as the lactazole A core.

More recently, Vinogradov and Suga reported a study on another dhAAR, FltJ_A_, which is a part of the lanthipeptide biosynthetic gene cluster in *Flavobacterium terrae*. Going beyond previous studies, this work uses mRNA display to determine enzymatic parameters (*k*_cat_/*K*_M_) of substrates in an approach they term DOMEK (mRNA-display-based one-shot measurement of enzymatic kinetics, Figure 17).

**Figure 17:**
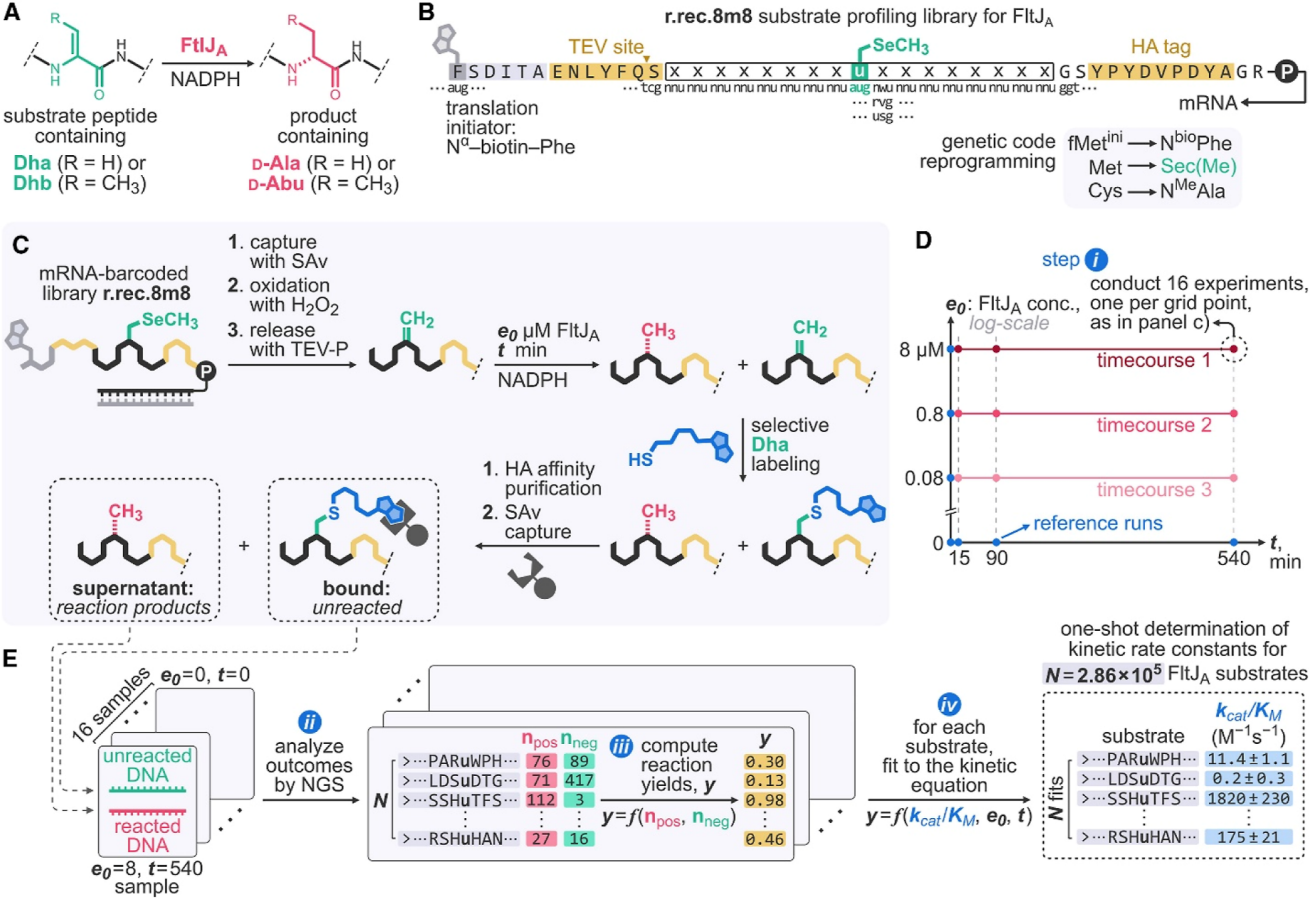
Reproduced from source publication^48^ (Figure 1 and caption therein) with permission from Elsevier. (A) FltJ_A_ catalyzes stereoselective reduction of dehydroamino acids in substrate peptides to d-amino acids using NADPH as a cofactor. In this study, all FltJ_A_ substrates contained Dha as the reactive amino acid. (B) Design of mRNA library r.rec.8m8, which encoded Dha-containing peptides as displayed. (C) Schematic workflow of a single mRNA display experiment. SAv, streptavidin. (D) Design of mRNA display-based time course experiments. In total, 16 pull-downs were conducted. (E) Schematic computational pipeline to determine *k*_cat_/*K*_M_ values from mRNA display data. Here, “u” is a single-letter code for Dha.

The overall library design used the same constant regions as previously, with subtle differences in the encoding and thus the final composition of the variable region. Translation was performed in the absence of Met and Cys, allowing for the reassignment of these codons to UAAs as described previously. For the library used in this study, the conserved Sec­(Me) was flanked by 8 variable residues with the overall sequence X8-U*-Z-X7, where X denotes NNU encoding, U* denotes Sec­(Me) encoded by AUG, and Z denotes a differently encoded variable position. Encoding at the Z residue was achieved with an 8:6:2 molar mixture of 3 sublibraries in which Z was encoded by NWU, RVG, and USG codons covering 8, 6, and 2 codons, respectively, so the 8:6:2 ratio ensures equal representation across the 16 codons covered. In combination, these codons cover 16 different amino acids but do not include encoding for Cys, Met, Pro, or Gln. In the previous study of MaeJ_C_ and WmnJ_C_, the 8 N- and C-terminal residues the equivalent library all used NNK encoding,^47^ which has the advantage of encompassing all 20 amino acids but disadvantages too, with codon bias and stop codons (more details on strategies to generate random libraries are covered in a dedicated section below). The rationale for the encoding choices are not explained within the article, but may have been to avoid multiple AUG codons, which are included within the NNK codon set and would correspond to multiple Sec­(Me) residues, though this was also the case in the previous study and not highlighted as a particular problem. Furthermore, a consideration noted by the authors was their aim to have a suitable library that sufficient sequencing depth of approximately 100-fold coverage can be achieved to get accurate results after the selection. To this end, they purposely sampled only ∼1 × 10^6^ sequences from the total X8-U*-Z-X7 pool (which itself has a theoretical diversity of 8 × 10^18^) by dilution and amplification by PCR.

Selections were conducted with varying FltJ_A_ concentrations (0, 0.08, 0.8, and 8 μM) and each sampled at multiple time points (0, 15, 90, and 540 min) corresponding to 16 selections in total, designed to cover a wide range of *k*_cat_/*K*_M_ values (Figure 17D and E). These were derived using a total quasi-steady-state approximation; for each peptide, the *k*_cat_/*K*_M_ value can be determined but not the individual parameters. The standard Michaelis–Menten equation is not applicable as individual displayed peptides are present at fM concentration, while the enzyme is in the μM range. Following sequencing, >280,000 peptides could be quantified across all 16 samples and the data fit to determine *k*_cat_/*K*_M_ values for each. Following extensive analysis, the authors concluded that they could accurately determine *k*_cat_/*K*_M_ values in the range 0.5–30,000 M^–1^ s^–1^ while estimates could be made for very good or very poor substrates outside this range. It was determined that the largest source of error arose from sampling effects during sequencing, and these could be overcome by sequencing in greater depth.

To validate their findings, 23 peptides were chemically synthesized, and the kinetic parameters (i.e., both *k*_cat_ and *K*_M_ individually) were determined for each peptide using traditional assays (termed “batch”). There was a strong correlation between the DOMEK and batch data (*R* ^2^ = 0.953), but the DOMEK values were typically 1.3- to 2-fold higher. Further insight was gained using reference-free analysis,^52^ revealing that in most cases, position-wise effects of amino acids are individual and additive (55% of the contribution from +1 position, >12% from −1 and +2 positions), but there were also some examples of cooperativity between amino acid positions. Despite the apparent simplicity of position-wise effects, the distribution of *k*_cat_/*K*_M_ values among the peptides revealed a highly complex multimodal fitness landscape.

In summary, RiPPs have received most of the recent attention due to their potential downstream applications as therapeutic compounds and could be viewed as occupying the overlap between the two mRNA-display paradigms; ligand discovery and enzyme characterisation. Accordingly, some of the most significant methodological advancement have arisen from studies on RiPPs; performing parallel ‘anti-selection’ campaigns and integrating with computational workflows has enable a greater depth of enzyme characterisation and make the most of the information available through NGS.

### Library Diversity

Throughout the articles reviewed here, numerous different ways to generate diversity within libraries have been used. Most rely on the use of degenerate codons to create large numbers of different peptides. The most common of these is the NNK mixture (N = A/C/G/U; K = G/U), which encompasses 32 different codons, encoding all 20 nAAs, but since there are more codons than amino acids, there is codon bias to consider. As shown in Table 2, with NNK codons, 3 amino acids are encoded by three codons, 5 are encoded by two codons, and 12 are encoded by one codon; a stop codon is also encoded, which could be problematic in some applications.

**Table 2:** Amino Acid Compositions Achieved through Different Degenerate Codon Regimes

	NNN	NNK	NNS	NNW	NNU	NWU/RVG/USG 8:6:2	Trimer Phosphoramidites^i^
Codons:	64	32	32	32	16	16	20
Average G/C content:	50%	50%	67%	33%	33%	44%	varies
Amino Acids:							
Ala	4	2	2	2	1	1	1
Arg	6	3	3	3	1	1	1
Asn	2	1	1	1	1	1	1
Asp	2	1	1	1	1	1	1
Cys	2	1	1	1	1	0	1
Gln	2	1	1	1	0	0	1
Glu	2	1	1	1	0	1	1
Gly	4	2	2	2	1	1	1
His	2	1	1	1	1	1	1
Ile	3	1	1	2	1	1	1
Leu	6	3	3	3	1	1	1
Lys	2	1	1	1	0	1	1
Met	1	1	1	0	0	0	1
Phe	2	1	1	1	1	1	1
Pro	4	2	2	2	1	0	1
Ser	6	3	3	3	2	1	1
Thr	4	2	2	2	1	1	1
Trp	1	1	1	0	0	1	1
Tyr	2	1	1	1	1	1	1
Val	4	2	2	2	1	1	1
**Total nAAs**:	**20**	**20**	**20**	**18**	**15**	**16**	**20**
**Stop**:	**3**	**1**	**1**	**2**	**0**	**0**	**0**

i- Trimer phosphoramidites can be deployed in various formats, we have assumed an equal mix of codons covering the 20 nAAs.

While conceptually more simple, single mutant variant libraries or libraries containing fixed amino acids at varying positions (e.g., the cysteine walk used with LynD) are less diverse and require more practical steps to achieve. For an 8-mer peptide, a saturating mutant approach (i.e. X8) can be achieved through a single primer with degenerate bases and have diversity of 20^8^ = 2.6 × 10^10^, whereas creating a library of saturation mutants at each position requires 8 separate primers and achieves many orders of less magnitude diversity: 8 × 19 = 152 (noting that at each position, one of the 20 amino acids will be the same as when that position is conserved in other sublibraries). However, small libraries can be more appropriate for certain applications, particularly in longer peptide sequences such as the smSVL library (570 different sequences) used to analyze pantocin mutants, which was 30 amino acids long. A saturating mutant library of this length would be too large to cover fully and could only be sampled at practical scales; indeed, a single copy of each possible 1.1 × 10^39^ sequences would require petamoles (10^15^ moles) of DNA.

Other encodings have been used in the papers discussed here: NNS (S = C/G) generates 32 codons, also covering all 20 nAAs and 1 stop codon with the same amino acid bias as NNK. The only difference lies in the codons used and their G/C content: 50% for NNK, 67% for NNS. Another encoding, NNW (W = A/U), also generates 32 codons, but only covers 18 nAAs, with Trp and Met missing, while also encoding 2 stop codons, making this an unusual choice. NNW codons were used by Iskandar et al. in conjunction with codon reprogramming, omitting Cys and Met from the translation reaction and supplementing with a flexizyme-charged tRNA so that Trp was encoded by UGU (usually a Cys codon). Thus, their selection covered 18 nAAs except Cys and Met, which are often disfavored anyway (at least for therapeutic ligand identification) due to their redox-active side chains.

NNU generates 16 codons, covering 15 nAAs, but with minimal bias (only Ser is encoded twice). In their DOMEK work, Vinogradov and Suga used a more elaborate approach by combining primers containing different degenerate codons: an 8:6:2 molar mixture of NWU, RVG, and USG. The overall amino acid profile covers 16 different amino acids without any bias and no stop codons. While more elaborate, this is straightforward to achieve practically, as primers can be simply mixed in a given ratio and treated as any other primer with degenerate bases. This approach is probably only viable for a single position as the number of primers required increases exponentially; e.g. 2 positions would need 9 primers, 3 positions need 27, etc. (every possible combination of NWU, RVG, and USG).

Finally, an alternative approach is to use trimer phosphoramidite building blocks during primer synthesis, which allows installation of intact codons as a single unit rather than a single nucleotide at a time, as was used by Zhu et al.^37^ This enables exquisite control over the amino acids included, which is the desired result for peptide display purposes without having to compromise on codon bias or undesirous in-frame stop codons. From a scientific point of view, there are no downsides to trimer phosphoramidites, but synthesis costs are >100-fold higher than for simple base mixtures (e.g., NNK), somewhat limiting their adoption.

## Trends

Across the reports surveyed here, there are some clear trends. The field has developed significantly over the past ∼25 years; the advent of massively parallel DNA sequencing has revolutionized the depth of information attainable from selection approaches and can be coupled with sophisticated computational analysis to give details simply unobtainable 25 years ago. Strikingly, of the 24 identified papers, 14 were published between 2020 and 2025: equivalent to 1 article approximately every 4 months (cf. one every 1.5 months for ligand discovery),^22,23^ indicative of the growing prevalence of mRNA-display being used across research laboratories. This could be partly due to the increased accessibility of next-generation sequencing, as well as developments to display technology to applications beyond ligand discovery.

Approaches to library design differ over time, with early campaigns typically using smaller, more focused libraries (<1 × 10^5^) and uncovering in the order of 100 hit sequences. More recent reports utilize degenerate codons to form fully randomized libraries of much larger diversity (>1 × 10^10^), one of the key strengths of in vitro display techniques, and can identify 10,000s of hits. Not all of these can usually be individually evaluated experimentally, but these data sets are appropriate for larger-scale computational analysis. Strategies also differ when a known residue is required, e.g., phosphorylation at Tyr, transglutaminase activity at Gln, or cyclodehydration at Ser/Cys; this is the case for 13 of the papers covered here (all except the proteases). In 10 of these reports, the target residue was encoded as an invariant AA within the library (denoted with a black outline throughout the figures) to ensure it is present in all members of the library. The other 3 papers (Abl-kinase, OGT, and STG) all rely on the occurrence of suitable codons in the randomized regions. Both approaches have yielded results, but perhaps the most obvious downside to relying on random occurrence is that much of the library will likely not even contain the correct amino acid, so the diversity sampled is lowered. On the other hand, this approach enhances the chance of serendipitous discoveries.

The use of additional modification steps varies significantly, likely simply a product of the requirements of a given enzyme: 3 articles use both pre- and post-enzymatic modification steps; 8 use a pre-enzymatic modification step only; 4 use a post-enzymatic modification step only; and 9 articles do not use either. In the latter category, 3 of these introduce UAAs during translation that are subsequently used in the capture step, and 3 others (all transglutaminases) utilize a modified co-substrate to add a capture handle in situ through the enzymatic reaction.

Biotin–streptavidin is the most common capture method, used by 18 papers with 3 using covalent immobilization and the final 3 relying on antibodies, all in the PTM section (note that we exclude antibodies to affinity tags, e.g., HA, as these have not been used to discriminate between modified and unmodified sequences). An advantage that most non-biotin recovery methods have is that they do not need pre- or post-enzymatic modification steps, but this may be offset by considerations around potential biases introduced. It is generally assumed that chemical modifications are completely sequence agnostic, but that may not always be the case, as highlighted by the periodate oxidation-resistant sequences identified in Vinogradov et al.’s dhAAR work.^47^

The introduction of UAAs is a key strength of mRNA-display, but is only used in 7 of the articles; 5 via flexizyme-mediated tRNA acylation, 1 through replacing an nAA with a surrogate, and 1 using amber codon suppression with an orthogonal tRNA aminoacyl synthetase. While initially surprising, many of the enzymes studied to date do not actually require UAAs for the selection procedures, which could be due to the techniques available when the research was conducted; more widespread adoption of UAA incorporation is a relatively recent development. From the approaches covered here, there are only three articles for which UAA incorporation was truly essential to study the enzymes in question: PDF, LanJ_C_s, and FltJ_A_, all published since 2020. In the other cases (and this is also true for LanJ_C_s and FltJ_A_), UAA incorporation was used to add a biotin-purification handle either directly or via subsequent derivatization, and theoretically, this could be achieved by other means, such as the use of an AviTag sequence (as done for identifying caspase substrates). Nevertheless, the introduction of UAAs as modified amino acids or precursors thereof does enable a far larger collection of enzymes to be studied with the approach and could be particularly useful to unpick combinations of PTMs, though this avenue is perhaps under-explored in work to date.

A key development is the adaptation of selection approaches to generate data suitable for more sophisticated computational workflows. Epitope tags in the peptide sequence allow secondary recovery of the unmodified sequences following the primary recovery of the modified sequences or vice versa. Recovery of both sets of sequences allows fitness calculations that are more reliable than just using enrichment from an assumed starting point. Control selections can also be performed to create background hits that can then be removed from the hit sequence to leave the true hits; in the case of WmnJ_C_, startlingly, >90% of hits were attributed to nonenzymatic activity, and yet conclusions could still be drawn from the remaining data after curating the data set. These approaches are not immediately applicable to ligand discovery, since that approach often generates “families” of peptides that may have different binding modes, thus preventing global analysis, though this may be applicable on sequences within an identified “family”.

While not always included in our summaries above to avoid excessive levels of detail, another trend is the number of purification steps used during these selections. Most reports include library purification steps removing residual components from in vitro translation, reverse transcription, etc. Some of the enzymes studied required these library purification steps to retain activity, while in other cases, improved results were seen with “cleaner” display pools. This is particularly important when isolating both substrate and non-substrate pools for computational analysis, as mentioned by Vinogradov et al.^44^ While the procedures used are clearly efficient enough for the selection to progress, none of the steps in a display selection will always proceed to 100%, so there may be mRNA unligated to the puromycin linker, unfused Pu-mRNAs, and/or free peptides in addition to the reaction components. Hence, without some purification/capture, it is not valid to assume that the “unbound” fraction is the same as the “unmodified” sequences and additional steps must be taken for that to be true. Fortunately, mRNA/cDNA complexes are very robust^58^ so various options for purification are available. Common epitope tags can be used for this purpose, though they need careful consideration; His_6_-tags are rarely used, likely as many commercially available translation kits (e.g., PUREexpress) contain components that are themselves His-tagged.^53^ Purification tags can be readily incorporated at the library design stage, and it is easy to assume that any additions will be benign and have no impact on the overall purpose of the selection, but this is not always the case. Shi et al. concluded that the C-terminal Pu-linkage was preventing the activity of OGT on displayed substrates^28^ and this is a fundamental constraint of mRNA-display; strategies for the inversion of the peptide oligonucleotide bond are limited unfortunately. An approach to overcome this has been demonstrated by Takatsuji et al. through multiple UAA incorporations, migrating the Pu-linkage site from the *C*-terminus to an amino acid side chain, enabling head-to-tail macrocyclization.^54^ One could envisage an adapted version of this workflow or a similar procedure involving cyclization followed by proteolytic cleavage to generate a free C-terminus while maintaining the mRNA-peptide linkage. There is at least one other strategy for achieving a similar outcome using a modified mRNA (without 5′ Puromycin) and a SNAP-tag encoded at the N-terminus of the peptide.^55^

## Future Prospects

A development to current approaches could be the extension to protein-sized molecules, addressing another key shortcoming of peptide-display approaches: a lack of higher order structure. Practically, this is straightforward, requiring only longer genes to be used, but consideration must be given to library size and quality control to check for folding. On the former matter, library sizes are effectively capped at ∼1 × 10^13^, the level at which only a single copy/molecule of a given sequence is present in experimental scales typically used in research laboratories. Larger libraries can be produced, but without increases in orders of magnitude to the scale of experiments (e.g., μLs to mLs), larger libraries cannot be covered completely and are only sampled. Whether this is a problem remains to be seen; with the capability of deep-learning models to extrapolate beyond their training data set, this may cease to be a concern.

Developments to nanopore sequencing, enabling direct sequencing of peptides, would be transformative and could effectively render display approaches redundant. A key issue with using mRNA-display to investigate modifications is the loss of information once a peptide is modified, as the mRNA is unchanged. Sequencing peptides directly could yield information about the cleavage site for proteases or the modification site for additive modifications. While it is currently possible under specific conditions to sequence peptides directly, the technology is not yet at the stage necessary to replace display approaches.^56,57^

As outlined in the preceding sections, the application of mRNA-display for substrate identification is still relatively new and underexplored. The availability of NGS and linking these data sets to computational approaches is an extremely powerful method to unravel complex substrate recognition regimes. Work to date has focused on relatively few enzyme classes but could be applied to any peptide-enzyme modifying enzyme, subject to the design of a suitable modification and capture strategy; diverse examples already exist in the literature as outlined in this article. To date, many have used the reactivity of sulfur with electrophiles as this represents, if not orthogonal, controllable reactivity, a key requirement. The mRNA-display approach has enormous potential to address many questions around enzyme selectivity, which is simply not possible by other means. There has been a surge in published reports in recent years (2020–2025), and it will be interesting to see whether this trend continues into the future.

## Notes

Figure 17 is a direct reproduction of Figure 1 from Vinogradov and Suga,^48^ “Measuring *k*_cat_/*K*_M_ values for over 200,000 enzymatic substrates with mRNA display” Chem, Volume 12, Issue 1, page 102737. Copyright (2025) with permission from Elsevier (agreement 6193041010579).

## Abbreviations

Ab: antibody
Abl: Abelson tyrosine
ADAM17: *Homo sapiens* disintegrin and metalloproteinase 17
AHA: Azidohomoalanine
anti-HA: tag antibody
biotin-PEG: biotinylated polyethylene glycol
BirA: biotin-(acetyl-CoA-carboxylase) ligase
Bt: biotin
C-Abl: *Homo sapiens* Abelson tyrosine kinase
cDNA: complementary DNA
CIB1: calcium and integrin-binding protein
Cov: covalent attachment
CuAAC: Cu­(I)-catalyzed alkyne–azide cycloaddition
Dha: dehydroalanine
dhAARs: dehydroamino acid reductases
DNA: deoxyribonucleic acid
dsDNA: double-stranded DNA
FRA3: Fragilysin isozyme 3
HA: HA-tag
HCV NS3/4A: hepatitis C virus NS3/4A protease
HPG: homopropargylglycine
IAA: iodoacetamide
LazBCDEF: the biosynthetic enzymes LazB, LazC, LazD, LazE, and LazF
LazDEF: LazD + LazE + LazF
LazF: nonspecific dehydratase
LC-MS: liquid chromatography–mass spectrometry
MAGE-4A: melanoma-associated antigen A4
MAP: methionine amino peptidase
megTYR: *Bacillus megaterium* tyrosinase
MPII: metalloproteinase II
mRNA: messenger RNA
nAAs: natural/proteinogenic amino acids
NGS: next-generation sequencing
O-GlcNAc: O-linked *N*-acetylglucosamine
OGT: human O-GlcNAc transferase OGT
PaaA: Pantocin biosynthesis enzyme
PAB: pentylamine-biotin
PCR: polymerase chain reaction
PDF: peptide deformylase
pPAF: *Para*-propargyloxy-l-phenylalanine
PTMs: post-translational modifications
Pu-mRNA: puromycin ligated mRNA
RFA: reference-free analysis
RiPP: Ribosomally synthesized, post-translationally modified peptide
RL2: O-linked *N*-acetylglucosamine (O-GlcNAc) monoclonal antibody
RNA: ribonucleic acid
Sec­(Me): methyl selenocysteine
smSVL: single variant library
TEV: Tobacco Etch Virus
TG: Transglutaminase
tRNA: tRNA
UAAs: unnatural amino acids/nonproteinogenic amino acids
UDP-GlcNAc: uridine diphosphate *N*-acetylglucosamine
v-Abl: Abelson murine leukemia virus tyrosine kinase
Xa: *Homo sapiens* factor Xa
Xaa: Randomized amino acid
α4G10: Phosphotyrosine-specific antibody.
DNA­(RNA) nucleotide mixtures: N = A/C/G/T­(U)
V: A/C/G
R: A/G
S: G/C
W: A/T­(U)
